# Supplementary material for: Spindle-locked ripples mediate memory reactivation during human NREM sleep
Source: Nat Commun. 2024 Jun 19;15:5249. doi: 10.1038/s41467-024-49572-8 (PMC11187142; doi:10.1038/s41467-024-49572-8)
Supplement: Supplementary file 1 — Supplementary Information [file 41467_2024_49572_MOESM1_ESM.pdf]

Supplementary Information

Spindle-locked ripples mediate memory reactivation during human NREM sleep

Thomas Schreiner[1], Benjamin J. Griffiths[1,2], Merve Kutlu[1], Christian Vollmar[3], Elisabeth Kaufmann[3], Stefanie Quach[4], Jan Remi[3], Soheyl Noachtar[3] & Tobias Staudigl[1]

1. Department of Psychology, Ludwig-Maximilians-Universität München, Germany.
2. Centre for Human Brain Health, University of Birmingham, United Kingdom.
3. Epilepsy Center, Department of Neurology, Ludwig-Maximilians-Universität München, Germany.
4. Department of Neurosurgery, University Hospital Munich, Ludwig-Maximilians-Universität München, Germany

Correspondence: Tobias.Staudigl@psy.lmu.de

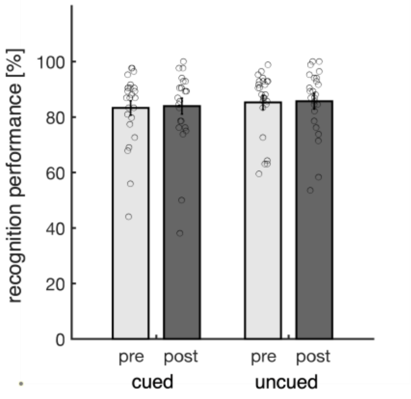

Supplementary Figure 1. Recognition memory performance (scalp EEG study, N = 25). Behavioral results for both experimental sessions pre- (light gray) and post-sleep (dark gray), separated into cued and uncued trials. Bar graphs show mean (±SEM) percentage of correctly recognized images ('hits'). Dots indicate individual memory performance of participants (N = 25). There was neither a significant main effect of test time as assessed by a repeated measures ANOVA ($F_{1,24} = 0.29$; $p = 0.59$), nor a significant interaction between test-time and cueing ($F_{1,24} = 0.08$; $p = 0.77$). Source data are provided as a Source Data file.

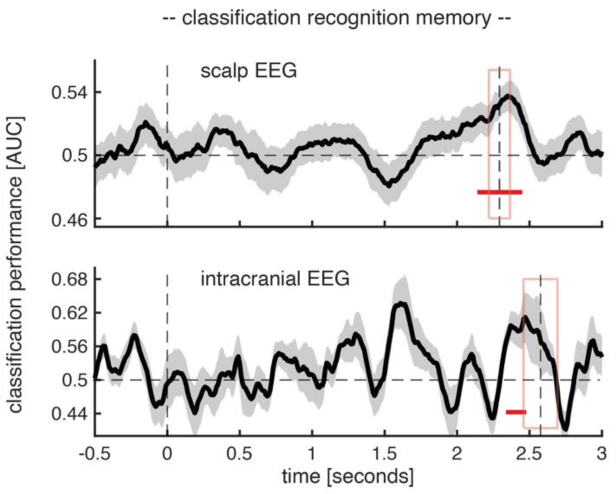

Supplementary Figure 2. Classification of later cued head orientations (left vs. right) locked to recognition onset (time = 0; first dashed vertical line). Later cued head orientations could be decoded (above chance) towards the end of recognition test trials, briefly preceding the onset of the associate prompt. The second vertical dashed line indicates the mean onset of the associative memory prompt. The red rectangle illustrates the standard error of the mean. The black solid line indicates decoding performance (±SEM). The horizontal dashed line indicates chance level performance (i.e., 0.5). The lower horizontal red line shows the temporal extent of significant decoding results as derived from a cluster-based permutation test, one-sided, corrected for multiple comparisons across time (scalp EEG study (N = 25) : p = 0.032; intracranial EEG study (N = 10): p = 0.043). Source data are provided as a Source Data file.

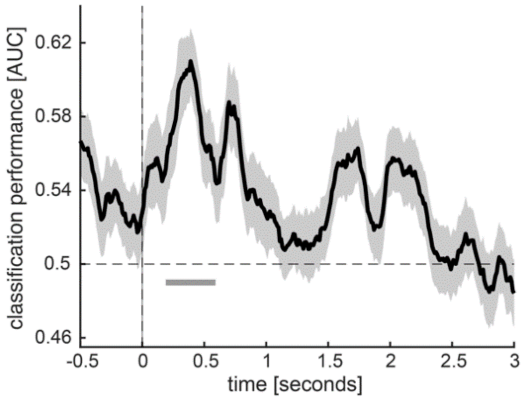

Supplementary Figure 3. Classification of later not cued head orientations during retrieval (scalp EEG study, N = 25). Later not cued head orientations (left vs. right) could be reliably decoded (above chance) from the retrieval data between 190 and 590 ms after the onset of the associate prompt (the black solid line indicates decoding performance (±SEM)). The horizontal dashed line indicates chance level performance (i.e., 0.5). The vertical solid line indicates the onset of associative retrieval trials (time = 0). The lower horizontal gray line shows the temporal extent of significant decoding results as derived from a cluster-based permutation test (two-sided, p = 0.006, corrected across time). Source data are provided as a Source Data file.

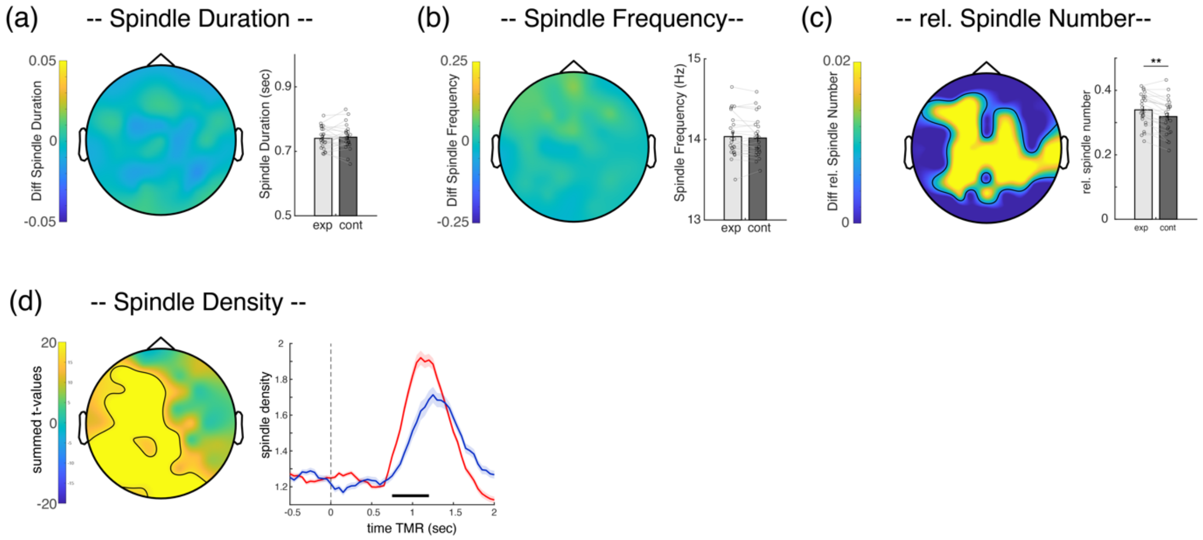

Supplementary Figure 4. Spindle characteristics scalp EEG (N = 25) (a) Spindle duration did not differ between conditions (experimental vs. control sounds) in the scalp EEG study (mean spindle duration experimental sounds: 0.7403 ± 0.0013 seconds, mean spindle duration control sounds: 0.7439 ± 0.0019, cluster-based permutation test, two-sided; p = 0.9, corrected across electrodes; the bars represent the mean spindle duration across all electrodes). (b) Spindle frequency did not differ between conditions (mean spindle frequency experimental sounds: 14.03 ± 0.008, mean spindle frequency control sounds: 14.01 ± 0.009, cluster-based permutation test, two-sided, p = 0.12, corrected across electrodes; the bars represent the mean spindle frequency across all electrodes). (c) The relative number of spindles (spindles emerging after cue onset in a time window between 0 and 1.5 seconds, normalized by the number of trials per condition), differed significantly between conditions (cluster-based permutation test, two-sided, corrected across electrodes) Experimental sounds were associated with more sleep spindles across frontal, central and parietal areas as compared to control sounds (relative spindle number experimental sounds: 0.32 ± 0.018, relative spindle number control sounds: 0.30 ± 0.019, p = 0.002, corrected across electrodes; the bars represent the mean relative spindle number across significant electrodes). (d) Comparing the occurrence probabilities of experimental sound (red) and control sound (blue) related spindles revealed higher spindle numbers for experimental sounds between 0.7 and 1.25 seconds after cue onset (cluster-based permutation test, two-sided, corrected for multiple comparisons, p = 0.002, corrected for multiple comparisons across time points and electrodes). Source data are provided as a Source Data file.

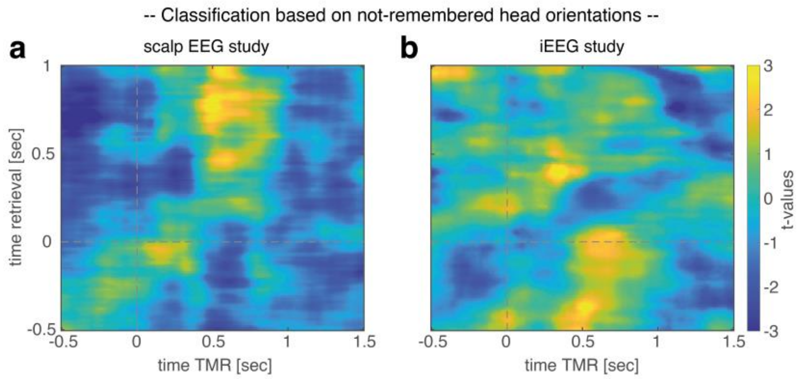

Supplementary Figure 5. Classification of not-remembered head orientations. Not-remembered head-orientations (left vs. right) were (a) neither decodable during TMR in the scalp EEG study (*p* = 0.14; cluster-based permutation test, two-sided, corrected for multiple comparisons, N = 25), (b) nor in the iEEG study (*p* = 0.88, cluster-based permutation test, two-sided, corrected for multiple comparisons, N = 10). Source data are provided as a Source Data file.

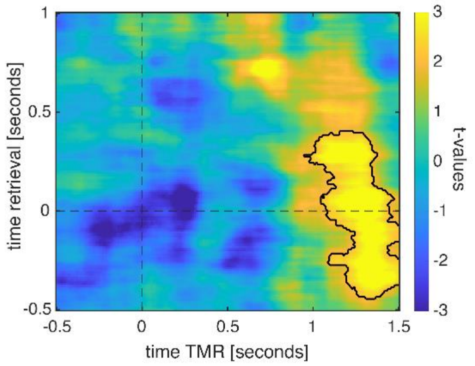

Supplementary Figure 6. Classification using TMR trials exhibiting increased levels of activity in the SO-spindle range (scalp EEG study, N = 25). Retrieval-related brain patterns (left vs. right head orientations) were reliably decodable during 'high power' TMR trials (*p* = 0.005; contour lines indicate the extent of the significant cluster as derived from a two-sided cluster-based permutation test, corrected for multiple comparisons; color range (blue to yellow) represents t values against chance level performance (i.e., 0.5)). Source data are provided as a Source Data file.

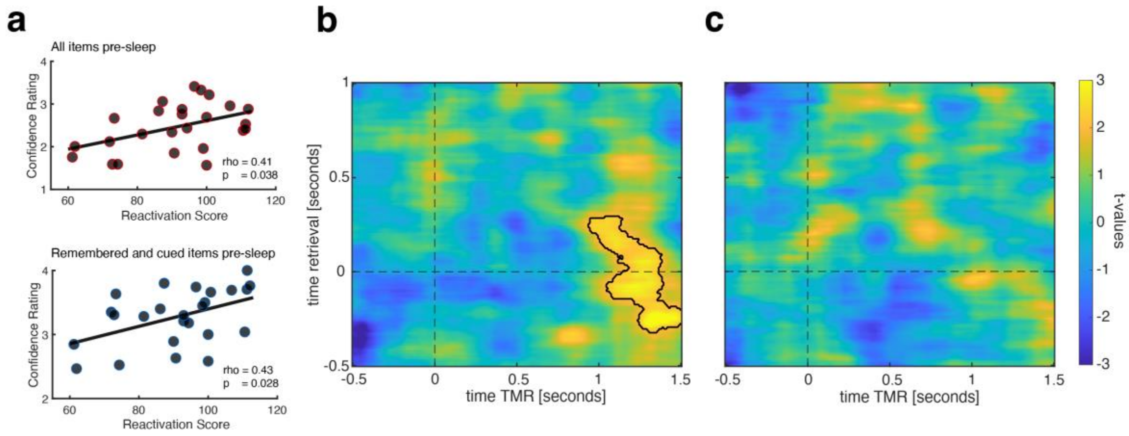

d

	Pre	Post	Pre-rem	Post-rem	Pre high conf. rem	Post high conf. rem
Confidence	2.5 ± 0.5	2.3 ± 0.6	3.2 ± 0.3	3.3 ± 0.2	54.39 ± 3.97 %	55.53 ± 3.99 %

Supplementary Figure 7. Relationship of confidence ratings and memory reactivation in the scalp EEG study (N = 25). In each retrieval trial, participants indicated how confident they were with their head orientation decision (scale from 0 corresponding to very uncertain to 4, very certain). (a) Participants' average confidence ratings across all trials correlated positively with the behavioral impact of TMR (i.e., reactivation score: relative difference from pre- to post-sleep for cued items - relative difference from pre- to post- sleep for uncued items * 100)/ pre-sleep remembered items; Spearman rho = 0.41; p = 0.038; N=25). Likewise, participants' average confidence ratings across all remembered and cued trials correlated positively with the behavioral impact of TMR (i.e., reactivation score: relative difference from pre-to post- sleep for cued items - relative difference from pre- to post- sleep for uncued items * 100)/ pre-sleep remembered items; Spearman rho = 0.43 p = 0.028; N=25). These results suggest that highly confident participants rather benefitted from TMR, while less confident participants exhibited a detrimental effect of TMR. (b) Classification using only high confidence trials (confidence rating = 4) as training data. Retrieval-related brain patterns (left vs. right head orientations) were reliably decodable when the decoder was trained on high confidence trials (p = 0.025; contour lines indicate the extent of the significant cluster as derived from a cluster-based permutation test, two-sided, corrected for multiple comparisons; color range (blue to yellow) represents t values against chance level performance (i.e., 0.5)). (c) Classification using lower confidence trials (confidence rating < 4) as training data. Retrieval-related brain patterns (left vs. right head orientations) were not decodable when the decoder was trained on lower confidence trials (p = 0.96, cluster-based permutation test, two-sided, corrected for multiple comparisons; color range (blue to yellow) represents t values against chance level performance (i.e., 0.5)). (d) Descriptives of confidence ratings: Means and SEM of confidence ratings of all pre-sleep trials [Pre], all post-sleep trials [Post], all remembered pre-sleep trials [Pre-rem] and all remembered post-sleep trials [Post-rem]. Percentage of high confidence trials of all remembered trials in the pre-sleep memory test [Pre high conf. rem] and percentage of high confidence trials of all remembered trials in the post-sleep memory test [Post high conf. rem]. Source data are provided as a Source Data file.

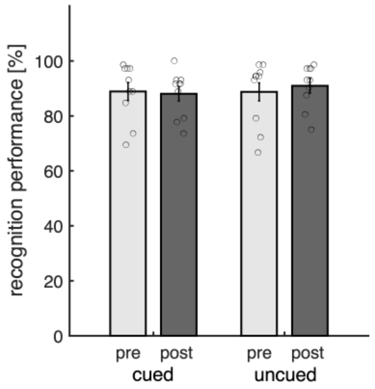

Supplementary Figure 8. Recognition memory performance (intracranial EEG study, N = 10). Behavioral results for both experimental sessions pre- (light gray) and post-sleep (dark gray), separated into cued and uncued trials. Bar graphs show mean (±SEM) percentage of correctly recognized images ('hits'). Dots indicate individual memory performance of participants (N = 10). There was neither a significant main effect of test time ($F_{1,9}$ = 0.06; p = 0.8) as assessed by a repeated measures ANOVA, nor a significant interaction between test-time and cueing ($F_{1,9}$ = 2.25; p = 0.16). Source data are provided as a Source Data file.

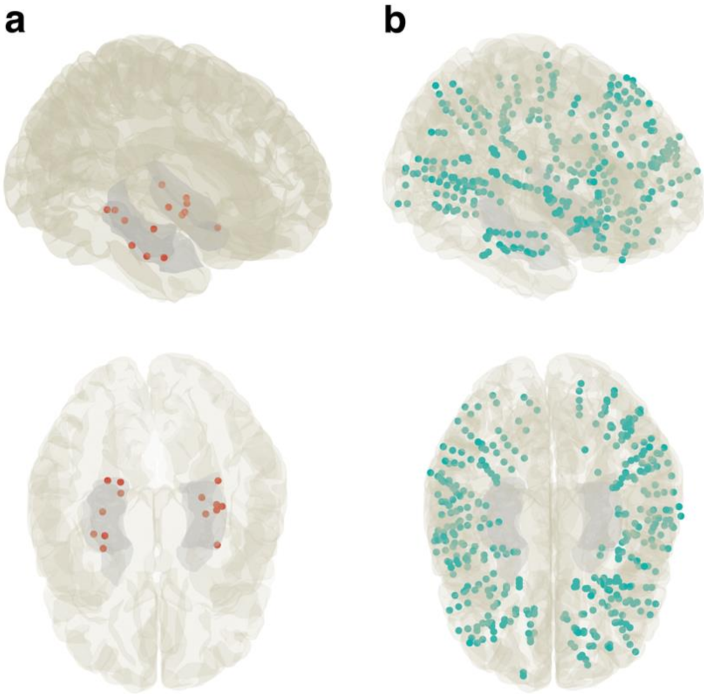

Supplementary Figure 9. iEEG electrode coverage (a) Group-level (N = 7) electrode coverage in MNI space of intracranial electrodes in the MTL (comprising contacts in the hippocampus, parahippocampus and entorhinal cortex; 14 contacts; red). (b) Group-level (N = 10) electrode coverage in MNI space of intracranial electrodes in non-MTL cortical areas (376 contacts, green). Source data are provided as a Source Data file.

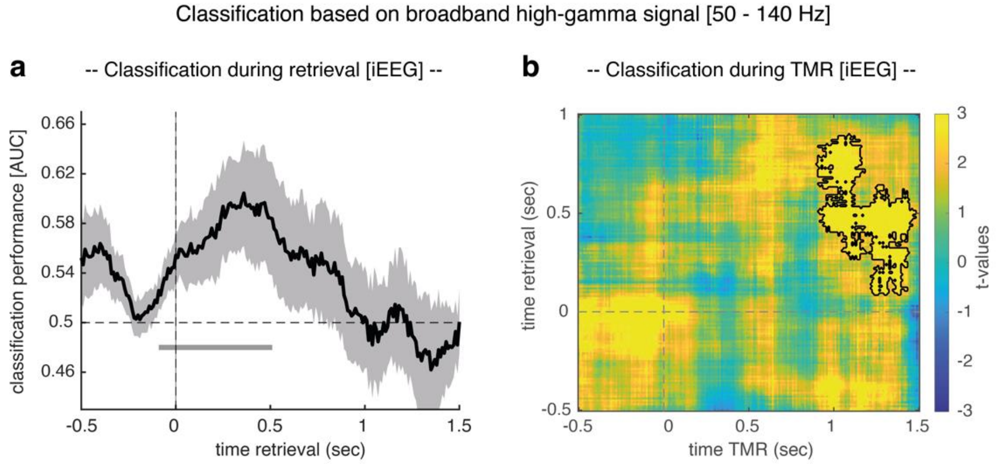

Supplementary Figure 10. Classification based in broadband high gamma (N = 10) (a) Later cued head orientation (left vs. right) could be reliably decoded (above chance) from the retrieval data filtered in the high-gamma range (50 – 150 Hz), starting around 80 ms after the onset of the associate prompt (the black solid line indicates decoding performance (±SEM across participants)). The horizontal dashed line indicates chance level performance (i.e., 0.5). The vertical solid line indicates the onset of associative retrieval trials (time = 0). The lower horizontal gray line shows the temporal extent of significant decoding results as derived from two-sided cluster-based permutation test (p = 0.019, corrected for multiple comparisons across time). (b) Head orientation-related brain patterns (left vs. right) were decodable during TMR based on high-gamma filtered data (50-150 Hz); contour lines indicate the extent of the cluster derived from a two-sided cluster-based permutation test (p = 0.022, corrected for multiple comparisons); color range (blue to yellow) represents t values against chance level performance. Source data are provided as a Source Data file.

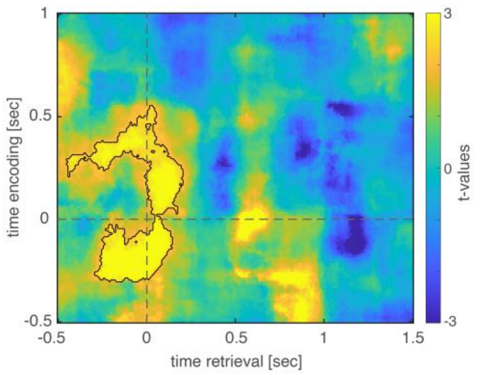

Supplementary Figure 11. Classification of encoding related brain activity during retrieval (iEEG study, N = 10). Encoding-related brain patterns (locked to image presentation; later cued left vs. right head orientations) were decodable during remembered retrieval trial (contour lines indicate the extent of the cluster as derived from a two-sided cluster-based permutation test, p = 0.0009 corrected for multiple comparisons; color range (blue to yellow) represents t values against chance level performance), indicating that indeed head orientation-related brain patterns were picked up by the classifier. Source data are provided as a Source Data file.

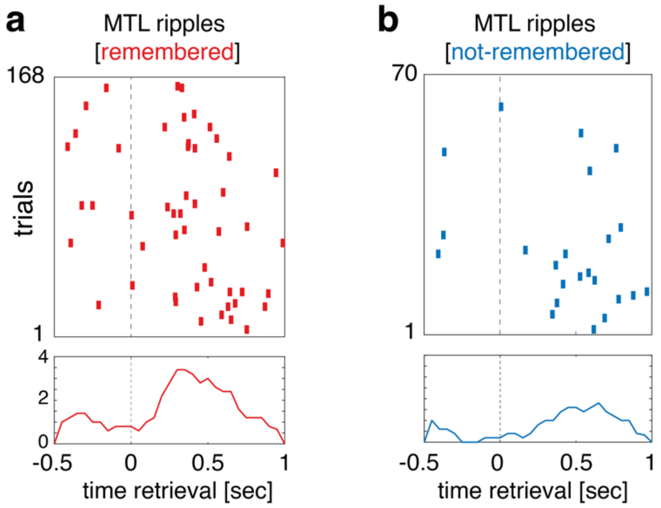

Supplementary Figure 12. Raster plots for retrieval-related MTL ripples. Exemplary raster plots for retrieval-related MTL ripples during remembered (a, red) and (b, blue) not-remembered trials. Bottom inserts illustrate the summed ripple numbers across time (bin size = 0.05 sec). Source data are provided as a Source Data file.

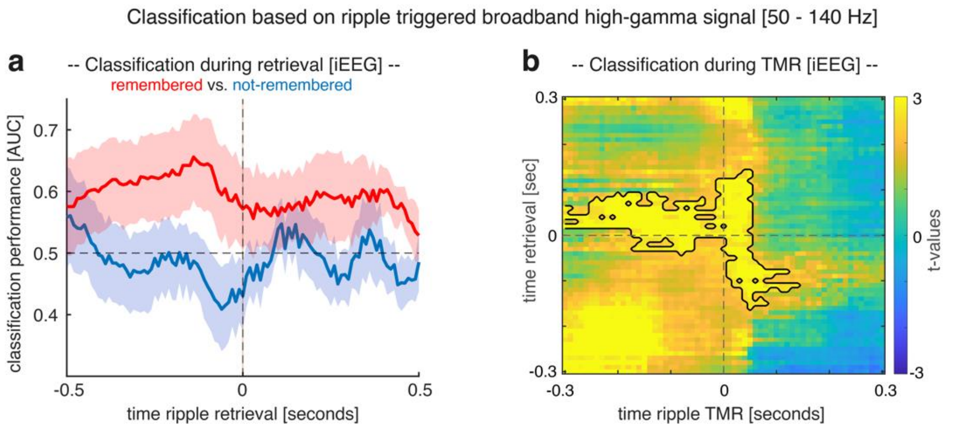

Supplementary Figure 13. Classification based on ripple triggered broadband high gamma (iEEG study, N = 5) (a) Retrieval-related classification of head orientations on basis of high-gamma filtered (50 to 150 Hz) iEEG segments centered around MTL ripples, specifically for remembered and not-remembered trials. While classification performance was generally higher for remembered as compared to not-remembered stimuli (peak of difference: -90 ms in relation to ripple center), this difference did not reach significance (two-sided cluster-based permutation test, p = 0.055, corrected for multiple comparisons across time, N=7). (b) Head orientation-related brain patterns (left vs. right) were decodable during the presence of MTL ripples based on high-gamma filtered iEEG data (50 – 150 Hz, contour lines indicate the extent of the significant cluster as derived from a two-sided cluster-based permutation test, p = 0.015 corrected for multiple comparisons, N=7; color range (blue to yellow) represents t values). Source data are provided as a Source Data file.

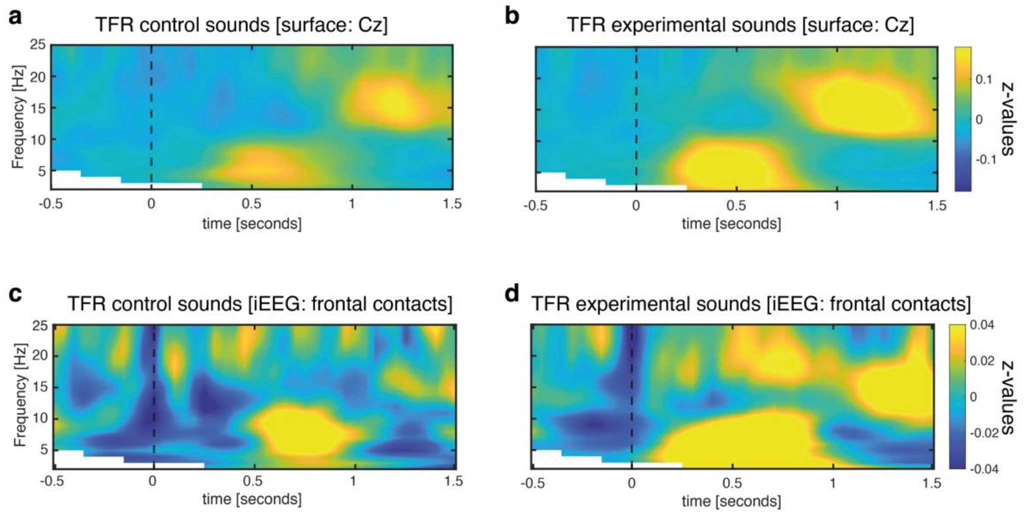

Supplementary Figure 14: TMR locked time–frequency representations (a + b) Average time–frequency representations for control sounds (a) and experimental sounds at electrode Cz (N = 25) (b) for the scalp EEG study. (c + d) Time–frequency representations for control sounds (c) and experimental sounds averaged across all frontal contacts (N = 156) (d) for the iEEG study. Source data are provided as a Source Data file.

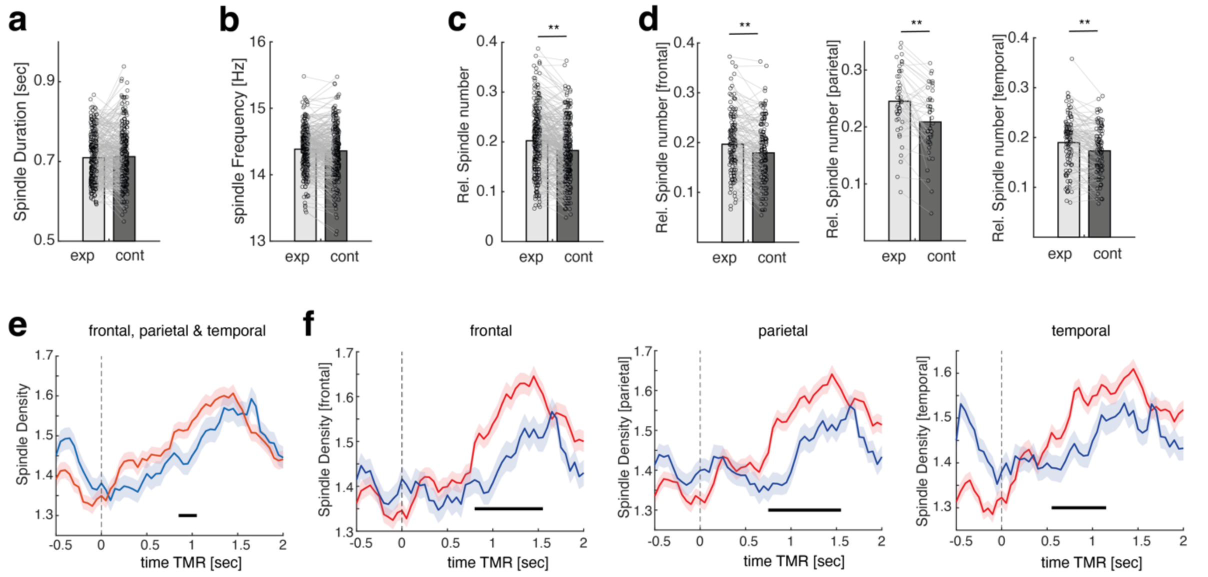

Supplementary Figure 15: Spindle characteristics iEEG (a) Spindle duration did not differ between conditions (experimental vs. control sounds) in frontal, parietal and temporal intracranial contacts (mean spindle duration experimental sounds: 0.709 ± 0.0032 seconds, mean spindle duration control sounds: 0.711 ± 0.0037, $t_{1,316}$ = -0.59, *p* = 0.55; the bars represent the mean spindle duration across all contacts (N = 317)). (b) Spindle frequency did not differ between conditions (mean spindle frequency experimental sounds: 14.38 ± 0.02, mean spindle frequency control sounds: 14.35 ± 0.01, $t_{1,316}$ = 1.77, *p* = 0.07). (c) The relative number of spindles (spindles emerging after cue onset in a time window between 0 and 1.5 seconds, normalized by the number of trials per condition), differed significantly between conditions. Experimental sounds were associated with more sleep spindles as compared to control sounds (relative spindle number experimental sounds: 0.201 ± 0.0033, relative spindle number control sounds: 0.181 ± 0.0034, $t_{1,316}$ = 8.16, p < 0.00001). (d) This effect became apparent in all examined regions (frontal contacts (N = 156): relative spindle number experimental sounds: 0.196 ± 0.0048, relative spindle number control sounds: 0.179 ± 0.0047, $t_{1,155}$ = 4.78, *p* < 0.00001; parietal contacts (N = 46): relative spindle number experimental sounds: 0.244 ± 0.0085, relative spindle number control sounds: 0.208 ± 0.0084, $t_{1,45}$ = 4.83, *p* = 0.000015; temporal contacts (N = 115): relative spindle number experimental sounds: 0.189 ± 0.0049, relative spindle number control sounds: 0.173 ± 0.0043, $t_{1,114}$ = 5.03, p < 0.00001). (e) Comparing the occurrence probabilities of experimental- (red) and control sound (blue) related spindles, revealed higher spindle numbers for experimental sounds between 0.85 and 1.1 seconds after cue onset across all frontal, parietal and temporal contacts (two-sided cluster-based permutation test, *p* = 0.024, corrected across time). (f) Occurrence probabilities of experimental sound related spindles were higher around 1 second post-stimulus in all examined regions (two-sided cluster-based permutation test; frontal: 0.8 – 1.5 seconds, *p* = 0.0009; parietal: 0.45 – 1.05 seconds, *p* = 0.0012; temporal: 0.65 – 1.05 seconds, *p* = 0.03; all corrected across time). Source data are provided as a Source Data file.

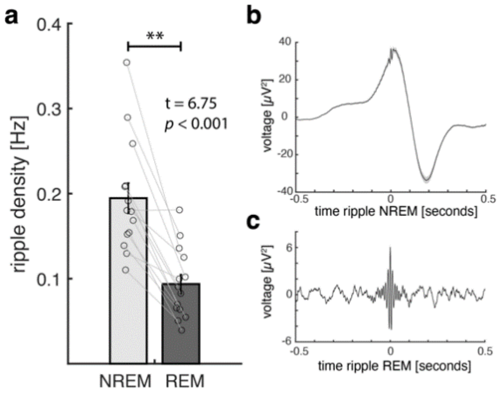

Supplementary Figure 16: Ripple density during NREM and REM sleep (iEEG study, N = 7) (a) Ripple density during NREM sleep was higher as compared to REM sleep (NREM: 0.19 ± 0.017 ripples per second; REM: 0.09 ± 0.011 ripples per second; $t_{1,14}$ = 4.81, two-sided dependent-samples t-test, *p* = 0.00001). (b) Ripple-centered grand average of all detected ripples during NREM sleep, locked to maximal negative amplitude (N = 3214 ± 393). (c) Ripple-centered grand average of all detected ripples during REM sleep, locked to maximal negative amplitude (N = 523 ± 54.18). Source data are provided as a Source Data file.

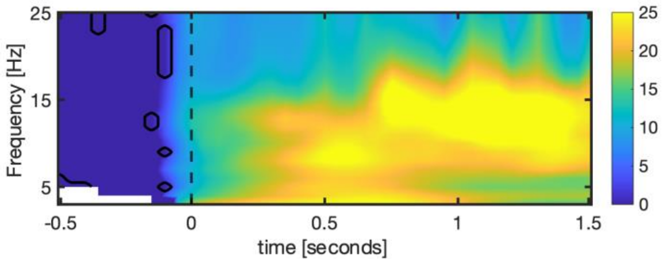

Supplementary Figure 17. Time–frequency representation difference map of high and low SO-spindle activity trials (iEEG study, N = 10). Oscillatory power was significantly higher for all time and frequency bins starting around cue onset for high power trials. Note the low frequency activity around 500 ms and subsequent spindle activity standing out (12-16Hz; two-sided cluster-based permutation test, $p < 0.00001$, corrected for multiple comparisons across time and frequency). Source data are provided as a Source Data file.

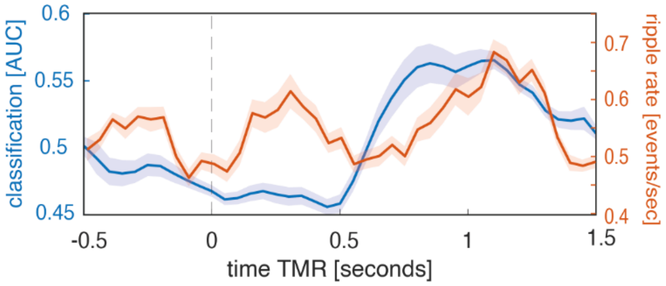

Supplementary Figure 18. Time course of ripple rate (events / sec) and reactivation signal (iEEG study). Blue: Classification output averaged across the relevant retrieval time [-150 to 200 ms; N = 10]. Red: Patient-averaged ripple rate across time ($N_{contacts}$ = 14). Source data are provided as a Source Data file.

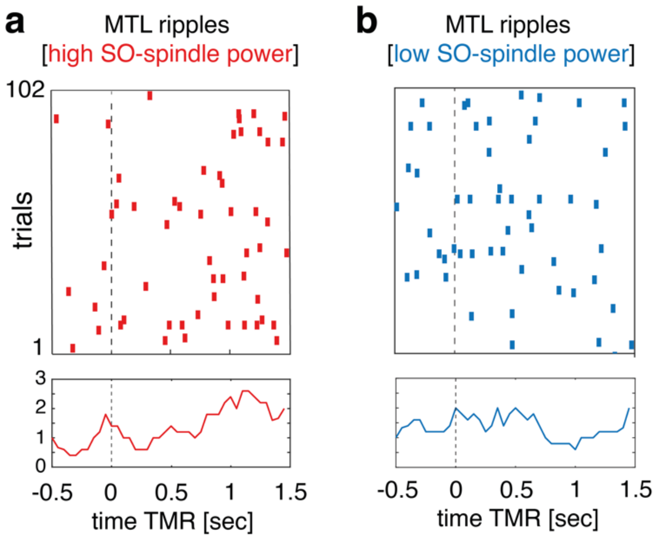

Supplementary Figure 19. Raster plots for NREM sleep MTL ripples (a) Exemplary raster plots for NREM sleep MTL ripples during high (**a, red**) and (**b, blue**) low SO-spindle power trials. Bottom inserts illustrate the summed ripple numbers across time (bin size = 0.05 sec). Source data are provided as a Source Data file.

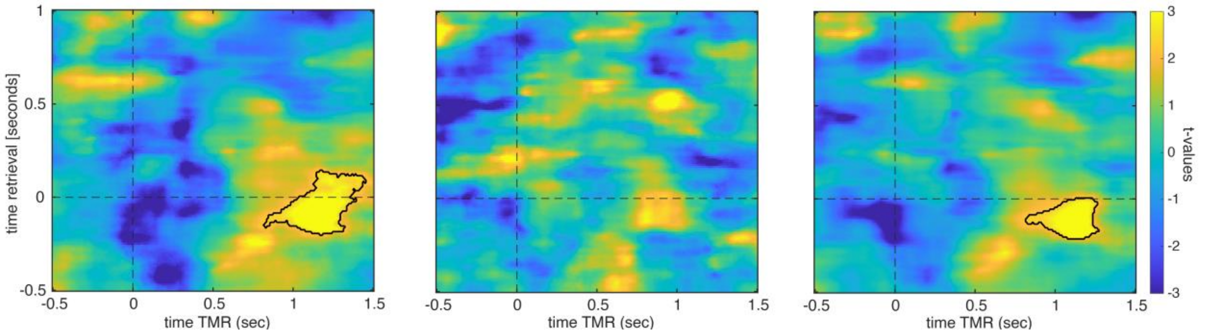

Supplementary Figure 20. (left) Classification using TMR trials exhibiting increased levels of activity in the SO-spindle range (iEEG study). Retrieval-related brain patterns (left vs. right head orientations) were reliably decodable during 'high power' TMR trials (contour lines indicate the extent of the significant cluster as derived from a two-sided cluster-based permutation test, p = 0.004 corrected for multiple comparisons, N = 10; color range (blue to yellow) represents t values against chance level performance (i.e., 0.5)). (middle) Classification using TMR trials exhibiting low levels of activity in the SO-spindle range (iEEG study). Retrieval-related brain patterns (left vs. right head orientations) were not decodable during 'low power' TMR trials (contour lines indicate the extent of the significant cluster as derived from a two-sided cluster-based permutation test, p = 0.71 corrected for multiple comparisons, N = 10; color range (blue to yellow) represents t values against chance level performance (i.e., 0.5)). (right) Classification using all TMR trials (irrespective of SO-spindle power; iEEG study). Retrieval-related brain patterns (left vs. right head orientations) were reliably decodable during 'high power' TMR trials (contour lines indicate the extent of the significant cluster as derived from a two-sided cluster-based permutation test, p = 0.047 corrected for multiple comparisons, N = 10; color range (blue to yellow) represents t values against chance level performance (i.e., 0.5)). Source data are provided as a Source Data file.

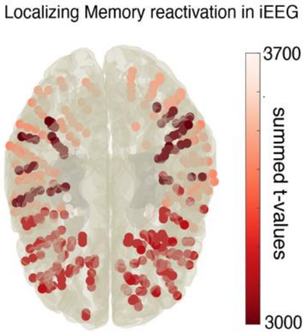

Supplementary Figure 21. Localizing memory reactivation in iEEG. To estimate the contribution of different brain areas to the cross-classification results (Fig. 4e), a leave-one-region-out approach was conducted. Specifically, we repeated the cross-classification 5 times and in each iteration iEEG contacts belonging to one region of interest (i.e., frontal, parietal, temporal, occipital, MTL) were excluded from the analysis. Results revealed that excluding temporal, occipital and MTL contacts did not affect the outcomes of the cross-classification procedure, given that head orientation-related activity was still decodable during TMR (without temporal contacts: p = 0.029, without occipital contacts: p = 0.026, without MTL contacts: p = 0.021; all p-values derived from two-sided cluster-based permutation tests, corrected for multiple comparisons). When excluding frontal or parietal contacts, no cluster of significant above chance classification became apparent (without frontal contacts: p = 0.054, without parietal contacts: p = 0.052; all p-values derived from two-sided cluster-based permutation test, corrected for multiple comparisons), indicating that these areas might have played a necessary role with regards to sleep based memory reactivation. It has to be noted that the results of the present analysis have to be interpreted with caution, as the number of contacts per region differed across patients. To visualize the obtained results, t-values within the cluster exhibiting the smallest p-value were summed. Hence, smaller numbers (color-coded in red) represent areas that putatively contributed to the detection of memory reactivation, since excluding them led to non-significant results. Source data are provided as a Source Data file.

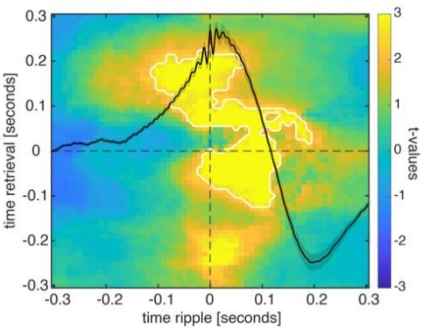

Supplementary Figure 22. Testing classification performance against chance level (iEEG study, N = 7). Retrieval-related brain patterns (left vs. right head orientations) were decodable during the presence of spindle-locked MTL ripples when tested against chance level at the sample level (i.e., 0.5; contour lines indicate the extent of the significant cluster, *p* = 0.007, two-sided cluster-based permutation test, corrected for multiple comparisons; color range (blue to yellow) represents t values). Source data are provided as a Source Data file.

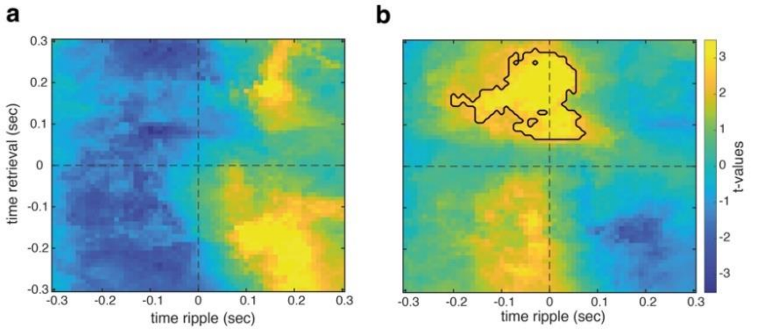

Supplementary Figure 23. Classification during uncoupled spindles (iEEG study, N = 7) (a) Retrieval-related brain patterns (left vs. right head orientations) were not decodable during the presence of uncoupled ripples (i.e., ripples without spindles; *p* = 0.073, two-sided cluster-based permutation test, corrected for multiple comparisons). (b) Head orientation-related brain patterns (left vs. right) were decodable during TMR when contrasting data segments centered on spindle-locked ripples and uncoupled ripples (contour lines indicate the extent of the cluster as derived from two-sided cluster-based permutation test, *p* = 0.032, corrected for multiple comparisons; color range (blue to yellow) represents t values against chance level performance). Source data are provided as a Source Data file.

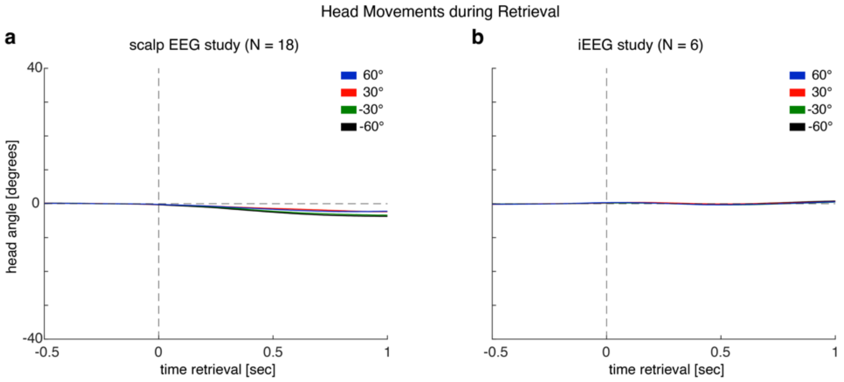

Supplementary Figure 24: Mean horizontal head orientation during associative memory retrieval prior to sleep. A head angle of 0° corresponds to participants fixating the center screen (during retrieval, participants were instructed to face the center screen, in contrast to the encoding phase, where they turned their gaze to the outer screens). Repeated-measures ANOVAs with 4 levels corresponding to the screen position where the item was presented during encoding (60°, 30°, -30°- -60°) did not show significant differences that survived correction for multiple comparisons during the time period from -0.5 sec to 1 sec (with respect to the onset of the associative memory prompt) in the (a) scalp EEG study (p= 0.16, two-sided cluster-based permutation test, corrected for multiple comparisons across time) nor in the (b) iEEG study (*p* = 0.13, two-sided cluster-based permutation test, corrected for multiple comparisons across time). Head position was available for 18 out of 25 participants in the scalp EEG study and 6 out of 10 patients in the iEEG study. Source data are provided as a Source Data file.

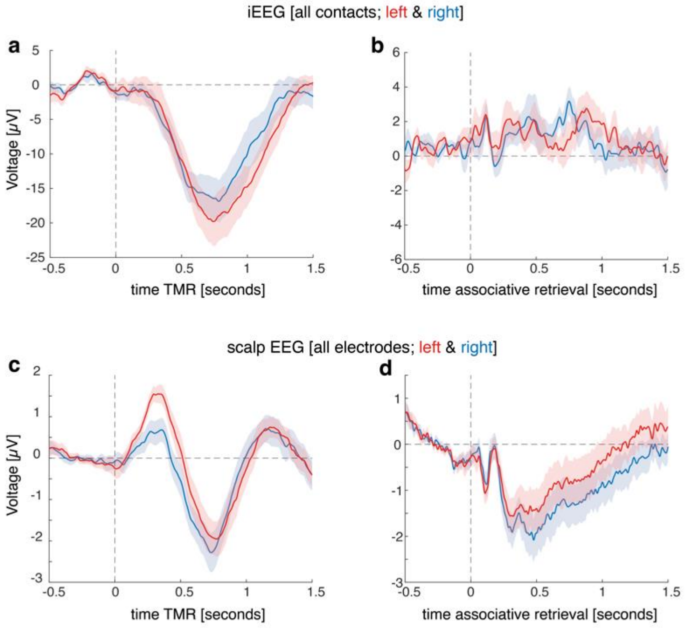

Supplementary Figure 25. (a + b) Event-related potentials (ERPs, mean ± SEM across participants) for the iEEG study (N=10) with regards to TMR (a) and the associative memory retrieval (b). The red waveform corresponds to ERPs associated with cues that were associated with left-sided head-orientations during encoding. The blue waveform corresponds to ERPs associated with cues that were associated with right-sided head-orientations during encoding. (c + d) ERPs (mean ± SEM across participants) for the scalp EEG study (N=25) with regards to TMR (c) and the associative memory retrieval (d). The red waveform corresponds to ERPs associated with cues that were associated with left-sided head-orientations during encoding. The blue waveform corresponds to ERPs associated with cues that were associated with right-sided head-orientations during encoding. Source data are provided as a Source Data file.

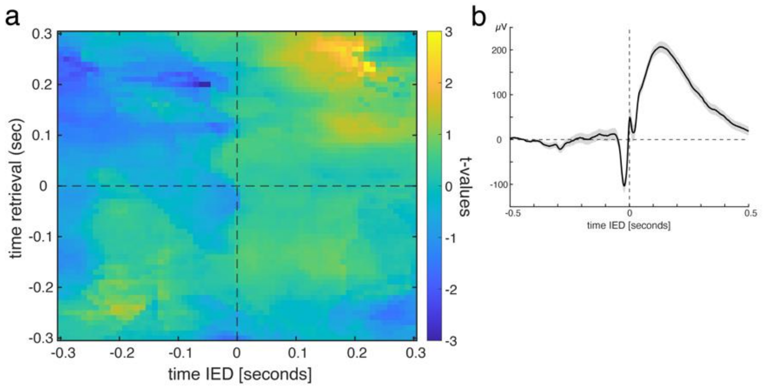

Supplementary Figure 26. Interictal epileptiform discharge (IED) triggered classification (iEEG study, N = 7). IEDs were automatically detected in MTL contacts of discarded data segments using established algorithms [17]. All intracranial EEG segments were centered around IEDs emerging 700 to 1400 ms after stimulus onset (i.e., same convention as in the ripple-triggered classification). (a) Training a classifier on the pooled associative retrieval data from both pre- and post-sleep sessions [−0.5 to 1s] and testing on the IED centered intracranial EEG data did not yield any significant result when tested against chance level performance (i.e., 0.5;p = 0.71, two-sided cluster-based permutation test, corrected for multiple comparisons). (b) Single subject example of IED centered ERP. Source data are provided as a Source Data file.

-- Ripple triggered classification [detection threshold 3SD; no artifact rejection] --

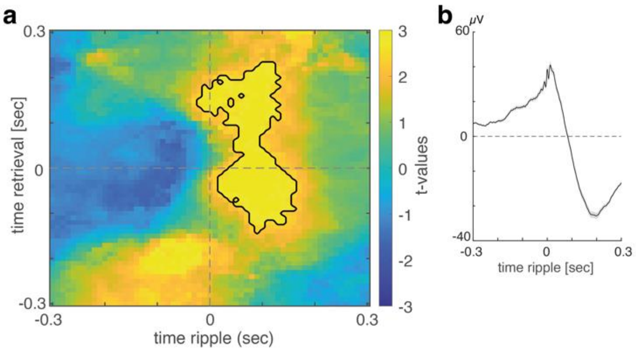

Supplementary Figure 27. (a) Classification procedure based on raw data (no artifact rejection), where ripples were detected using a threshold of 3 SD. Head orientation-related brain patterns (left vs. right) were decodable during the presence of spindle locked MTL ripples (contour lines indicate the extent of the significant cluster as derived from two-sided cluster-based permutation test, p = 0.032, corrected for multiple comparisons; color range (blue to yellow) represents t values). (b) Ripple-triggered grand average of all detected ripples (7 patients, 14 contacts; locked to maximal negative amplitude) during TMR (-.5 to 1.5 seconds; 49.86 ± 6.06 ripples in 308 ± 17.05 trials). Source data are provided as a Source Data file.

	N1	N2	SWS	REM	WASO	TST [min]
Sleep stage [%]	4.1 ± 0.5	46.8 ± 1.4	22.6 ± 1.0	21.0 ± 1.2	0.04 ± 0.01	421.4 ± 9.7

Supplementary Table 1. Sleep characteristics EEG study. Data are means ± s.e.m. N1, N2: NREM sleep stages N1 & N2, SWS: slow-wave sleep, REM: rapid eye movement sleep, WASO: wake after sleep onset. Source data are provided as a Source Data file.

	N1	N2	SWS	REM	WASO	TST [min]
Sleep stage [%]	4.1 ± 0.9	44.9 ± 2.7	20.6 ± 2.5	23.1 ± 2.0	5.2 ± 1.7	480.3 ± 31.4

Supplementary Table 2. Sleep characteristics iEEG study. Data are means ± s.e.m. N1, N2: NREM sleep stages N1 & N2, SWS: slow-wave sleep, REM: rapid eye movement sleep, WASO: wake after sleep onset. Source data are provided as a Source Data file.

P_1	Parietal
P_2	Temporal
P_3	Parietal
P_4	Parietal
P_5	Frontal
P_6	Frontal
P_7	Frontal

Supplementary Table 3. Location of cortical contact exhibiting the strongest power in the spindle band (12-15 Hz)
