# Supplementary material for: Spindle-locked ripples mediate memory reactivation during human NREM sleep
Source: Nat Commun. 2024 Jun 19;15:5249. doi: 10.1038/s41467-024-49572-8 (PMC11187142; doi:10.1038/s41467-024-49572-8)
Supplement: Supplementary file 3 — Reporting Summary [file 41467_2024_49572_MOESM3_ESM.pdf]

nature portfolio

Corresponding author(s): Tobias Staudigl

Last updated by author(s): May 27, 2024

Reporting Summary

Nature Portfolio wishes to improve the reproducibility of the work that we publish. This form provides structure for consistency and transparency in reporting. For further information on Nature Portfolio policies, see our Editorial Policies and the Editorial Policy Checklist.

Statistics

For all statistical analyses, confirm that the following items are present in the figure legend, table legend, main text, or Methods section.

n/a	Confirmed	
☐	☒	The exact sample size (*n*) for each experimental group/condition, given as a discrete number and unit of measurement
☐	☒	A statement on whether measurements were taken from distinct samples or whether the same sample was measured repeatedly
☐	☒	The statistical test(s) used AND whether they are one- or two-sided<br>*Only common tests should be described solely by name; describe more complex techniques in the Methods section.*
☐	☒	A description of all covariates tested
☐	☒	A description of any assumptions or corrections, such as tests of normality and adjustment for multiple comparisons
☐	☒	A full description of the statistical parameters including central tendency (e.g. means) or other basic estimates (e.g. regression coefficient) AND variation (e.g. standard deviation) or associated estimates of uncertainty (e.g. confidence intervals)
☐	☒	For null hypothesis testing, the test statistic (e.g. *F*, *t*, *r*) with confidence intervals, effect sizes, degrees of freedom and *P* value noted<br>*Give P values as exact values whenever suitable.*
☒	☐	For Bayesian analysis, information on the choice of priors and Markov chain Monte Carlo settings
☒	☐	For hierarchical and complex designs, identification of the appropriate level for tests and full reporting of outcomes
☐	☒	Estimates of effect sizes (e.g. Cohen's *d*, Pearson's *r*), indicating how they were calculated

Our web collection on statistics for biologists contains articles on many of the points above.

Software and code

Policy information about availability of computer code

Data collection	The experimental paradigm was conducted using Matlab and the toolbox 'Psychtoolbox' (http://psychtoolbox.org/).<br>Scalp EEG was recorded using EEGo mylab (ANT Neuro, Enschede, Netherlands).<br>Intracranial EEG was recorded using XLTEK Neuroworks software (Natus Medical, San Carlos,<br>562 California, US) and an XLTEK EMU128FS amplifier.
Data analysis	MATLAB 2022a ,Mathworks, https://uk.mathworks.com/;<br>Fieldtrip Toolbox v.09/01/2020, http://www.fieldtriptoolbox.org/;<br>CircStat Toolbox v.1, https://www.jstatsoft.org/article/view/v031i10;<br>MVPA light toolbox (https://github.com/treder/MVPA-Light)<br><br>Custom code is available at Ludwig- Maximilians-Universität via https://data.ub.uni-muenchen.de/487/.

For manuscripts utilizing custom algorithms or software that are central to the research but not yet described in published literature, software must be made available to editors and reviewers. We strongly encourage code deposition in a community repository (e.g. GitHub). See the Nature Portfolio guidelines for submitting code & software for further information.

Data

Policy information about availability of data

All manuscripts must include a data availability statement. This statement should provide the following information, where applicable:

- Accession codes, unique identifiers, or web links for publicly available datasets
- A description of any restrictions on data availability
- For clinical datasets or third party data, please ensure that the statement adheres to our policy

Data acquired from the healthy participants are available at Ludwig- Maximilians-Universität via https://data.ub.uni-muenchen.de/487/. Due to privacy laws, data acquired from the patients are not openly available, though (subject to privacy laws) can be provided by contacting the corresponding author. Source data are provided with this paper.

Research involving human participants, their data, or biological material

Policy information about studies with human participants or human data. See also policy information about sex, gender (identity/presentation), and sexual orientation and race, ethnicity and racism.

Reporting on sex and gender	No sex- or gender-based analyses were conducted as we had no a priori reason to suspect sex-/gender-related differences in our experiment.
Reporting on race, ethnicity, or other socially relevant groupings	No race-, ethnicity- or other socially relevant-based analyses were conducted as we had no a priori reason to suspect race-/ ethnicity/other socially relevant-related differences in our experiment.
Population characteristics	See "Behavioural and social sciences study design" section
Recruitment	The healthy participants were recruited using a university-based e-mail list of interested individuals and through Facebook adverts. Potential sources of bias could be present in that individuals who were interested in research and / or had sufficient time may be more likely to self-select and volunteer for the study. Patient recruitment involved asking those undergoing the relevant surgery to take part following the implanation of depth electrodes (Epilepsy Center, Department of Neurology, Ludwig-Maximilian Universität, Germany).
Ethics oversight	The study was approved by the local ethics committees at LMU (Department of Psychology & Medical Faculty of the Ludwig).

Note that full information on the approval of the study protocol must also be provided in the manuscript.

Field-specific reporting

Please select the one below that is the best fit for your research. If you are not sure, read the appropriate sections before making your selection.

☐ Life sciences ☒ Behavioural & social sciences ☐ Ecological, evolutionary & environmental sciences

For a reference copy of the document with all sections, see nature.com/documents/nr-reporting-summary-flat.pdf

Behavioural & social sciences study design

All studies must disclose on these points even when the disclosure is negative.

Study description	The study had a quantitative within-participants experimental design
Research sample	Healthy participants were 25 young adults (mean age: 25.2 ± 0.6; 16 female). This population was sampled primarily due to availability for sleep- session commitment. 10 Patients (7 female; age: 31.20 ± 3.46) took part in the experiment. This population was exclusively sampled due to availability in the clinical context.
Sampling strategy	The sampling procedure was self-selecting: participants were contacted with details about the study and asked to get back in touch if they were interested in taking part. Sample size was set to 25 participants based upon previous targeted memory reacitvation studies studies (e.g., Cairney et al., 2018; Schreiner et al., 2015). The sample size of the patient study was solely driven by the supply of patients and is in line with other studies involving intracranial recordings (e.g., Topalovic et al., 2023, Nat. Neuro., n=12; Stangl et al., 2020, Nature, n=5).
Data collection	The paradigm was presented on standard computer monitors. Electrophysiological data was collected using an ANT Neuro EEG system or (in the case of the patients) Spencer depth electrodes on a XLTEK Neuroworks system.
Timing	Data collection began around June 2020 and concluded around December 2022
Data exclusions	Fourteen healthy participants had to be excluded due to insufficient sleep or technical problems. Four patients had to be excluded due to technical difficulties.

Non-participation	No participants / patients dropped out/declined to participate.
Randomization	N/A - it was a within-participant design, so participants were not assigned to experimental groups

Reporting for specific materials, systems and methods

We require information from authors about some types of materials, experimental systems and methods used in many studies. Here, indicate whether each material, system or method listed is relevant to your study. If you are not sure if a list item applies to your research, read the appropriate section before selecting a response.

Materials & experimental systems

n/a	Involved in the study
☒	☐ Antibodies
☒	☐ Eukaryotic cell lines
☒	☐ Palaeontology and archaeology
☒	☐ Animals and other organisms
☒	☐ Clinical data
☒	☐ Dual use research of concern
☒	☐ Plants

Methods

n/a	Involved in the study
☒	☐ ChIP-seq
☒	☐ Flow cytometry
☒	☐ MRI-based neuroimaging

April 2023
