# Supplementary material for: Spindle-locked ripples mediate memory reactivation during human NREM sleep
Source: Nat Commun. 2024 Jun 19;15:5249. doi: 10.1038/s41467-024-49572-8 (PMC11187142; doi:10.1038/s41467-024-49572-8)
Supplement: Supplementary file 2 — Peer Review File [file 41467_2024_49572_MOESM2_ESM.pdf]

nature portfolio

# Peer Review File

## Spindle-locked ripples mediate memory reactivation during human NREM sleep

**Open Access** This file is licensed under a Creative Commons Attribution 4.0 International License, which permits use, sharing, adaptation, distribution and reproduction in any medium or format, as long as you give appropriate credit to the original author(s) and the source, provide a link to the Creative Commons license, and indicate if changes were made. In the cases where the authors are anonymous, such as is the case for the reports of anonymous peer reviewers, author attribution should be to 'Anonymous Referee' followed by a clear attribution to the source work. The images or other third party material in this file are included in the article's Creative Commons license, unless indicated otherwise in a credit line to the material. If material is not included in the article's Creative Commons license and your intended use is not permitted by statutory regulation or exceeds the permitted use, you will need to obtain permission directly from the copyright holder. To view a copy of this license, visit http://creativecommons.org/licenses/by/4.0/.

**Editorial Note**: This manuscript has been previously reviewed at another journal that is not operating a transparent peer review scheme. This document only contains reviewer comments and rebuttal letters for versions considered at Nature Communications.

REVIEWER COMMENTS

Reviewer 1:

The authors report evidence from two experiments using targeted memory reactivation (TMR) to elicit learning-related signals, one of which involved scalp EEG and one intracranial EEG. Interestingly, unlike many studies showing TMR memory benefits, they found a TMR deficit in both experiments. Also in both experiments, they found learning-related signals that overlapped with other physiological signals (SOs and spindles) linked with successful memory retention. The novel finding here is that the intracranial recordings allowed them to also link ripples from the medial temporal lobe with these learning-related signals.

Overall, the paper is well-written and the presentation of the results are clear. However, in my opinion, it is not a great fit for this journal for two reasons. First, while the behavioral anti-TMR effect is interesting, the connection between the neural results and behavior is unclear (see some questions about it below). This connection seems imperative for a journal focused on behavior. Second, while this is an impressive undertaking obtaining two studies, including one with patient iEEG data, the main novel result – that MTL ripples temporally overlap with other learning-related reactivation signals – are results that almost had to be true, given that prior findings had already linked ripples with spindles (e.g., Siapas & Wilson, 1998; Staresina et al., 2015; Jiang et al., 2019) and spindles with reactivation (e.g., Jiang et al., 2017; Wang et al., 2019; Schechtman et al., 2023). I am not discounting that it is important to verify things linked theoretically by various papers, but this makes it hard to consider the paper a major conceptual advance required for publication in NHB. I would try eLife or PLoS Biology. Nevertheless, I recognize editors / other reviewers may disagree, so here are my other concerns / suggestions:

Major concern:

As stated above, I believe the authors are largely missing a connection between neural and behavioral results here. First, I should state that the negative TMR effects are interesting! But I think it remains unclear mechanistically why they occurred, and they also make one curious about what the neural results mean considering this lack of clarity. Do these reactivation signals reflect something that helps its related memories, hurts them, or does both at the same time? The authors did some exploratory analyses along these lines in Supplementary Figure 12 that I found interesting, but they also did not include this in Results section. I suppose here I am at risk of encouraging the authors to over-explore their data, but perhaps something could come from these analyses. Questions: First, which of the two experiments did these Fig S12 data come from? Second, is this effect replicable across experiments? The authors could pre-register their prediction for the other experiment right now, test this, and include the other experiment as being predicted (based on the other experiment). Third, is there a relationship between overall pre-sleep memory performance

and reactivation score? Fourth, did the authors try any other analyses linking behavioral and neural results that it would be useful to know were not significant?

Medium concern:

The nature of the reactivated representation seems like it has a lot of overlap with lateralized motor movements during encoding and lateralized motor imagery during wakeful retrieval, considering that the participants are physically moving their heads towards the appropriate screen on their left vs. right. For this reason, I found it surprising the authors omitted prior sleep investigations on lateralized motor imagery (Kouider et al., 2014; Andrillon et al., 2016, 2017) and a study showing these signals emerge after TMR (Wang et al., 2019). The latter study is also relevant because they also showed that these reactivation signals increased with spindles, which is a core finding here (Lines 132-137; Lines 224-228). Additionally, did the authors investigate or control for possible head movements during wakeful retrieval?

Minor concerns:

Line 51 – Were participants given feedback after the pre-sleep test?

Line 70 – I believe the authors meant to have ‘cued’ here instead of ‘uncued’.

Lines 87-9 – Great explanation! I was wondering about this while looking at the figure, but this helps explain why there is reactivation before t = 0.

Line 118 – “TMR-induced”

Figure 2 – Please define ‘TFR’ somewhere before one encounters it in the figure. It is 'time-frequency representation', right?

Line 209 – “TFR-related”

Figure 3 – Did I miss something, or is there less of the anticipatory (pre-time 0) reactivation signal here? Is this just random noise or is there a difference in the design somehow?

Line 285 & Line 345 – “instrumental” and “driving” are examples of causal language where correlational language is warranted. Please be mindful of this throughout the paper.

Line 342 – many researchers now avoid the word ‘transfer’ with respect to systems consolidation or add nuance that this is likely inexact, incomplete, or memories are instead transformed (such as proposed in Multiple Trace Theory or especially the later Trace Transformation Theory). Please consider changing the wording.

Line 363-5 – Please fix this sentence. I believe they are using ‘unbeknownst’ unusually or inappropriately. I believe the authors mean to say that it is important to consider that, in prior studies showing spindle effects, there could additionally be ripple effects which went undetected due to the lack of appropriate recording method to detect them, but I am not sure.

Line 402 – Comma after “memory”

Line 403 – “deteriorated”

Line 527 – “was comprised of” -> “comprised”

Reviewer 2:

The study investigated the role of spindle-locked ripples in memory reactivation during human NREM sleep, in two distinct and complementary populations: healthy volunteers (scalp EEG) and patients with implanted electrodes (intracranial EEG). In both cases, participants learned a list of names visually presented at one specific spatial location (one out of five screen monitors horizontally displayed), together with the specific sound associated with each of the four non-central positions. They thus learned both the names to be recognised later amongst distractors, and the association between the name and its spatial location and the associated sound. During the subsequent NREM sleep episode in a TMR procedure, they were presented (cued) with two out of the four sounds (one left- and one right-side association), with the prediction that specific cueing would improve memory performance for the associated material. Unexpectedly, behavioral results showed the opposite pattern, that is performance decreased overnight both for cued and uncued material, but more for the cued than for the uncued material, suggesting an interference effect due to the targeted stimulation. The effect was similar both in healthy volunteers and patients, thus quite robust. At the neural level, MVPA classification during NREM sleep was above chance level to

differentiate left- and right-side related cues, as compared to a neutral sound never presented during the learning episode. In healthy subjects, higher levels of SO-spindle activity were associated with increased classification performance, an effect interpreted by the authors as reflecting the promotion of the read-out of TMR-induced memory reactivation. Furthermore, in patients, intracerebral recordings of spindle-locked ripples in the medial temporal lobe highlighted a significant association with classification performance, suggesting that the temporal locking between spindles and ripples within slow association of NREM sleep is instrumental for memory reactivation to unfold (authors' interpretation).

This is an original, very well-designed and written study, with sound methods and analyses, that certainly contributes to our understanding of the associations between spindles, ripples and slow oscillations in the context of information processing. Notwithstanding, I have some reservationd about the interpretation of these results in terms of memory reactivation processes, given that all reported effects and associations are obtained with classification performance, which seems to be at least implicitly considered by the authors as a complete surrogate of memory processing. However, they neglect here the fact that behavioural memory performance on the next day was actually worse after cueing than non-cueing, which may cast doubts about the functional relevance of the associations that have been found. In my view, this apparent contradiction needs to be addressed in a more detailed way than it is currently done in the discussion, and claims about the unfolding of memory reactivation processes during NREM sleep in relation with TMR should be toned down in favour a more cautious interpretation. To be clear, I like here very much the work done and do not contest its significance at the neurophysiological level, but have more doubts about the functional interpretation in terms of memory consolidation processes.

Besides this major comment, here belowother remarks:

1. the way the paper is written suggests that memory consolidation would take place only during sleep, which is exaggerated. E.g., see the first line of the abstract, at least it should be said that memory consolidation PARTIALLY relies on the reactivation of previous experience during sleep; or the first line in the introduction which is also ambiguous in this respect.

2. page 4, behavioural results: please clarify how memory performance is computed. Based on the claim in the last sentence that there is no effect for recognition memory, I assume that it is the association between the name and its location, but it needs to be better clarified. Also, in the sentence lines 69-70, it is said that uncued items are better remembered than uncued items .... Probably a typo

3. Lines 78-81, could you explain why pulling together bot pre- and post-sleep session to extract head-orientation related neuronal activity during retrieval ? Would it not be more appropriate to use only pre-sleep activity as it is expected that post-sleep activity would be modulated by the night TMR manipulation ?

4. Learning task: it is said that the screens are positioned at – or + 30° and – or + 60 °, does that correspond to the visual angle ? What was the screen to eye distance and the size of the monitors ?

5. As mentioned above, assuming that cued-related MVPA classification performance is equivalent to memory processing performance is an analogical assumption; this should be better discussed and grounded by the authors.

6. It is said (e.g. lines 218-219) that SO-spindle activity coordinates temporal occurrence of ripples , but the association is with TFRs that do not have high temporal resolution. Would it be possible for instance to try to time-lock the beginning of the ripples with the beginning of the spindles ?

Reviewer 3:

The study investigates the relationship between memory reactivation during sleep and the occurrence of ripple-spindle complexes, using the Targeted Memory Reactivation (TMR) paradigm.

Despite a slight deterioration in memory performance post-TMR, the results effectively elucidate the association between memory reactivation and the timing of ripple/spindle complexes during sleep and suggest an interesting link between confidence rating and the TMR effect. This is a novel contribution that significantly advances the field and will be of interest to a large audience.

Overall, the paper is well-written, and the results are conveyed clearly and effectively. Nevertheless, to make the results more compelling and to rule out alternative explanations, the authors need to address several critical issues and conduct additional analysis, as detailed below:

Major points –

1) While the authors do illustrate a significant enhancement in the slow oscillation (SO) and spindle power, it would be valuable to include raw spectrograms that visibly depict spindle activity during the different TMR conditions. Merely showcasing power increases represents just one facet of the analysis; it is crucial to consider additional spindle characteristics as well (duration, spatial distribution, and mean frequency).

2) Is there a difference in the spatial distribution of spindles between experimental and control sounds in the iEEG data? such analysis will strengthen the argument for functionally distinct ripples/spindles induced by the TMR cues.

3) The authors should include figures showing the spectrotemporal signature of ripples and spindles in their data, as well as 2-3 example traces. As detailed in Liu et al (https://www.nature.com/articles/s41467-022-33536-x) – the authors should report ripple rate across the different sleep stages, to demonstrate what we already know about ripples, namely, that ripple rate is generally higher during non-REM sleep compared to REM. This will further convince the readers that the authors detected genuine physiological ripples.

4) To facilitate comparison with analogous studies examining awake human ripples (Vaz et al., 2019; Norman et al., 2019, 2021; Henin et al., 2021; Dickey et al., 2022; Kunz et al., 2023;), the authors should present the data in Fig 3e and Supplementary Figure 8 in terms of ripple rate (events/sec), and include a single trial visualization of ripples relative to the retrieval and TMR cues (either ripple raster or ripple density showing pooled-trials × time).

5) The authors should cite the papers mentioned above as they have direct relevance to these results, showing ripple-mediated memory reactivation during wakefulness.

6) The method employed for ripple detection may not be optimal. It is advisable for the authors to thoroughly review relevant intracranial studies on human hippocampal ripples published within the last five years (including the papers mentioned above), and subsequently fine-tune their methods accordingly. For instance, it is recommended to use a more stringent ripple detection threshold of 3-4 SD and exclude candidate ripples that occur near inter-ictal epileptic discharges and transient muscular artifacts that appear on the EMG electrode, rather than excluding 36% of the trials due to putative epileptiform activity. The authors seem to discard valuable information by implementing this stringent data selection criterion (i.e., effectively excluding ~36% of trials). Do the results hold true when trials are not subjected to this particular selection process?

7) Critical point: the authors should extend the analysis in Figure 3e also to the awake retrieval phase. Does retrieval exhibit a comparable increase in ripple rate in response to the associative cue, as shown by Vaz et al. (2019)? If so, will the authors find stronger ripples activation for correctly remembered items?

This analysis will substantially strengthen the link between the detected ripples and the mnemonic process.

8) Critical point: the authors should add a figure showing the cross-classification performance using the broadband high-gamma intracranial signal. This signal reflects neuronal activation more accurately and directly, compared to the raw iEEG signal. It is crucial to investigate whether the timing of reactivation remains consistent between the raw iEEG and high-gamma signals, as this has significant implications for interpreting the results.

9) The authors should run the high-gamma analysis of ripple-triggered activity both during TMR trials and awake retrieval trials. If ripples indeed play a role in re-activating memory associations, they should do so both during sleep and awake retrieval (as shown by previous studies). The authors should examine if the similarity between the cortical peri-ripple patterns induced by TMR and those induced by successful memory retrieval, is significantly higher compared to peri-ripple patterns induced by foil presentation (where memory associations do not exist).

General comments:

10) Overall, the findings don't conclusively establish whether the observed reactivation genuinely indicates a mnemonic process or whether it primarily pertains to activity linked with cue identity. Put differently, can the authors discern whether the reactivated representations are connected to memory representation, head orientation, or the identity of auditory cues? Specifically, it would be beneficial if the authors could show that a classifier trained on encoding trials (when subjects actually moved their head), is able to decode the same head-orientations during retrieval.

(If this analysis proves inconclusive, it is advisable for the authors to temper their claims in the discussion regarding reactivation of representational content that is specifically tied to "head-orientation"; currently the results do not conclusively show that)

11) Line 416: “we found a positive correlation between memory confidence in the pre-sleep memory test and the effect of TMR...” – this is an important finding. As a follow-up analysis, it would be beneficial for the authors to investigate whether the behavioral outcomes and their correlation with the TMR effect become more pronounced when excluding "guesses." These would be trials where subjects reported very low confidence levels (<3). If subjects do not remember specific items during the pre-sleep test, it is not expected that TMR would substantially enhance their memory. The potential for memory enhancement through TMR would primarily come into play when a memory trace already exists.

12) Multivariate analysis: how did you choose to retain 30 principal components? how much variance these components explain? Please include the ERPs associated with the two head-orientation classes for readers who want to appreciate the quality of the EEG signal.

13) Where is the iEEG reactivation effect localized? The authors are encouraged to provide a cortical map illustrating the spatial distribution of electrodes that play a significant role in the classification result.

Minor points:

14) Line 39: “establishing a role of sharp wave-ripples for memory reactivation…” - the role of ripples in memory reactivation has already been fairly established in previous works, especially during awake retrieval; the present work only extends this to sleep and specifically to ripples that are nested within spindles; this work is certainly not the first to show that ripples are related to memory reactivation (e.g., Zhang et al. 2018).

15) Line 75: “Head orientation-related activity is reactivated…” – unless the authors show that the reactivation was specifically related to head orientation representations, the authors should revise their claims and refer to this activity as memory-prompt related activity.

16) Line 84: the authors refer to a figure that does not exist (Fig 1d).

17) Line 115-116: what is meant by – “please note that statistical tests were done for illustrative purposes only”? are all the statistics in this figure are for “illustrative purposes”?

18) Line 468: typo, remove the word ‘and’.

19) Line 342, 385: the authors should also cite Rothchild et al 2016 (https://www.nature.com/articles/nn.4457) showing TMR in rodents. The authors should discuss their results considering this paper – which suggests that auditory TMR facilitates generation of memory-related ripples by biasing hippocampal activity.

20) Lines 652, 690: “Fig 3c” –> should be fig 3d

21) Fig 1d: correct “TFR” to TMR

22) In all figures – please indicate what the error bars represent (SEM across subjects/patients/electrodes/trials?)

**Reviewer#1:**
**Remarks to the Author:**

The authors report evidence from two experiments using targeted memory reactivation (TMR) to elicit learning-related signals, one of which involved scalp EEG and one intracranial EEG. Interestingly, unlike many studies showing TMR memory benefits, they found a TMR deficit in both experiments. Also in both experiments, they found learning-related signals that overlapped with other physiological signals (SOs and spindles) linked with successful memory retention. The novel finding here is that the intracranial recordings allowed them to also link ripples from the medial temporal lobe with these learning-related signals.
Overall, the paper is well-written and the presentation of the results are clear. However, in my opinion, it is not a great fit for this journal for two reasons. First, while the behavioral anti-TMR effect is interesting, the connection between the neural results and behavior is unclear (see some questions about it below). This connection seems imperative for a journal focused on behavior. Second, while this is an impressive undertaking obtaining two studies, including one with patient iEEG data, the main novel result that MTL ripples temporally overlap with other learning-related reactivation signals are results that almost had to be true, given that prior findings had already linked ripples with spindles (e.g., Siapas & Wilson, 1998; Staresina et al., 2015; Jiang et al., 2019) and spindles with reactivation (e.g., Jiang et al., 2017; Wang et al., 2019; Schechtman et al., 2023). I am not discounting that it is important to verify things linked theoretically by various papers, but this makes it hard to consider the paper a major conceptual advance required for publication in NHB. I would try eLife or PLoS Biology. Nevertheless, I recognize editors / other reviewers may disagree, so here are my other concerns / suggestions:

**Major concern:**

As stated above, I believe the authors are largely missing a connection between neural and behavioral results here. First, I should state that the negative TMR effects are interesting! But I think it remains unclear mechanistically why they occurred, and they also make one curious about what the neural results mean considering this lack of clarity. Do these reactivation signals reflect something that helps its related memories, hurts them, or does both at the same time? The authors did some exploratory analyses along these lines in Supplementary Figure 12 that I found interesting, but they also did not include this in Results section. I suppose here I am at risk of encouraging the authors to over-explore their data, but perhaps something could come from these analyses. Questions: First, which of the two experiments did these Fig S12 data come from? Second, is this effect replicable across experiments? The authors could pre-register their prediction for the other experiment right now, test this, and include the other experiment as being predicted (based on the other experiment). Third, is there a relationship between overall pre-sleep memory performance and reactivation score? Fourth, did the authors try any other analyses linking behavioral and neural results that it would be useful to know were not significant?

<u>Response:</u> We agree with the reviewer that the detrimental effects of TMR on behavior may seem unexpected at first sight. However, as outlined in detail in the discussion (page 15, line 443), our results are very much in line with a growing number of recent studies reporting detrimental effects of TMR on behavior (e.g., Oyarzún et al., 2017; Antony et al., 2018; Witkowski et al., 2021; Joensen et al., 2022). All of these studies, including ours, have one feature in common: several targets were associated with one memory cue, which led to the suggestion that if associations between multiple targets and one cue vary in strength, TMR might elicit the reactivation of targets most strongly associated with the cue (Joensen et al., 2022). As mentioned by the Reviewer, in our exploratory analysis (Supplementary Fig. 23), we found a positive correlation between memory confidence in the pre-sleep memory test and the effect of TMR, which might indicate that TMR may have mainly reactivated targets that were

strongly associated with their cues and further strengthened their association, while weaker associations were outcompeted.
To answer the Reviewer's first question, the data in Supplementary Fig. 12 come from the scalp EEG study. As described in the methods section (page 19, line 604), no confidence rating was obtained for the iEEG study due to constraints in the clinical context, impeding the possibility to replicate this result across experiments.

We made this point now clearer in text (page 15, line 458):

*…Interestingly, in an exploratory analysis (see Supplementary Fig. 23), we found a positive correlation between memory confidence in the pre-sleep memory test and the effect of TMR in the scalp EEG study (due to time constraints no confidence rating was obtained in the iEEG study)…*

That said, we do not think that the detrimental effect of TMR on behavioral measures contradicts the assumption that TMR-induced memory reactivation during NREM sleep, as picked up by the used classification procedures.

First, TMR did have a robust effect on memory performance, across participants and independent studies (scalp and iEEG), indicating that TMR exerted a systematic influence on neural processes during NREM sleep. We would like to point out here that we replicated our behavioral results (and the main EEG results) in an independent sample, which is exceptionally rigorous compared to most of the literature in the TMR field.

Second, as pointed out above and discussed in the manuscript, our results are very much in line with recent work reporting detrimental effects of TMR on behavior (e.g., Oyarzún et al., 2017; Antony et al., 2018; Witkowski et al., 2021; Joensen et al., 2022).

Third, our classification results in both the scalp EEG and the iEEG study, indicating that prior learned stimulus categories were decodable during the presence of sleep spindles, are very much in line with previous work showing TMR-induced but also endogenous memory reactivation during related time windows (i.e., connected to the presence of sleep spindles) using similar classification procedures (e.g., Cairney et al., 2018; Schreiner et al., 2021; Ngo and Staresina, 2022). This consistency across independent studies, especially in the case of studies where TMR exhibited beneficial effects on behavior (Cairney et al., 2018; Ngo and Staresina, 2022), provides further indication that our TMR procedure induced memory reactivation but with an opposite consequence on behavior. Finally, the obtained cross-classification results were highly specific with regards to retrieval processes. Retrieval-related neural patterns of head-orientations could only be successfully classified during TMR in case of correctly remembered head-orientations (see Supplementary Fig. 5 for cross-classification based on not-remembered trials and below), while decodable time windows with regards to the retrieval data highly overlapped between the cross-classification and within retrieval classification procedures. We added this argumentation to the discussion (page 15, line 466):

*…In sum, several lines of argument support the assumption that our classification procedure indeed picked up neural signatures of memory reactivation, despite the fact that TMR exerted a detrimental influence on memory performance. First, TMR did have a robust effect on behavior, both across participants and independent studies (scalp and iEEG), indicating a systematic influence on neural processes during NREM sleep. Second, as outlined in detail above, a growing number of studies reported similar detrimental effects on behavior when several targets were associated with a given TMR cue [30,78,79,85]. Third, multivariate analysis methods (such as MVPA [86] or representational similarity analysis [87]) have been proven valuable tools to identify memory reactivation during both wakefulness and sleep [5,77]. Our cross-classification results, indicating that prior learned stimulus categories were decodable during the presence of sleep spindles, are in line with previous work reporting TMR-induced or endogenous memory reactivation locked to spindles (e.g., [52,86–88]). This consistency in terms of the neural conditions (i.e., the presence of sleep spindles) favoring decodability is also found with studies where TMR exhibited beneficial effects on behavior [87,88], which further supports the notion that our TMR*

*procedure indeed induced memory reactivation, but with opposite consequences on behavior. Finally, the obtained cross-classification results were highly specific with regards to retrieval processes. Retrieval-related neural patterns of head-orientations could only be successfully classified during TMR in case of correctly remembered head-orientations (see Supplementary Fig. 5 for cross-classification based on not-remembered trials), while decodable time windows with regards to the retrieval data highly overlapped between the cross-classification and within retrieval classification procedures...*

In addition, as suggested by the reviewer we now correlated pre-sleep memory performance and the reactivation score in both studies, but did not find any significant relationship in either of the studies (scalp EEG study: r = -0.04, *p* = 0.83; iEEG study: r = 0.39, *p* = 0.26). Beyond that, we did not conduct any additional non-significant analyses linking behavioral and neural results that were not reported.

**Medium concern:**

The nature of the reactivated representation seems like it has a lot of overlap with lateralized motor movements during encoding and lateralized motor imagery during wakeful retrieval, considering that the participants are physically moving their heads towards the appropriate screen on their left vs. right. For this reason, I found it surprising the authors omitted prior sleep investigations on lateralized motor imagery (Kouider et al., 2014; Andrillon et al., 2016, 2017) and a study showing these signals emerge after TMR (Wang et al., 2019). The latter study is also relevant because they also showed that these reactivation signals increased with spindles, which is a core finding here (Lines 132-137; Lines 224-228). Additionally, did the authors investigate or control for possible head movements during wakeful retrieval?

Response: We agree with the reviewer that the mentioned studies (Kouider et al., 2014; Andrillon et al., 2016, 2017; Wang et al., 2019) are highly interesting, but we do not feel that there is large conceptual overlap to the present study. The studies mentioned by the Reviewer have in common that they leveraged the lateralized readiness potential (LRP), which is thought to reflect motor preparation on a certain body side (Schurger et al., 2021), in order to assess (i) the extent to which the brain continues to process external stimuli (Kouider et al., 2014; Andrillon et al., 2016), (ii) the capability of the sleeping brain to form implicit memories (Andrillon et al., 2017) or enhance (iii) the decodability of memory reactivation during NREM sleep (Wang et al., 2019). Hence, they used body movements to track sleep-related cognitive processing with the help of the LRP. In contrast to this, the scope of our study was the opposite, as we aimed for investigating whether head orientations would act as spatial context in episodic memory (please see discussion, page 14, line 408). Hence, we did not use head movements as a tool to reveal sleep-based processing, but instead, head orientations were the object of investigation. In addition, we are not aware of studies showing an LRP for head movements. While the LRP has been shown to reflect brain activity related to motor activity of certain body parts (i.e., arm, leg or foot) (Shibasaki and Hallett, 2006), movement of the head, as part of a gaze shift, like in the present study, is under the control of the oculomotor system (i.e., FEF, Superior Colliculus (Cowie and Robinson, 1994; Sparks et al., 2001; Elsley et al., 2007)).
While we would still refrain from incorporating the work of Kouider et al., 2014; Andrillon et al., 2016, 2017 in our manuscript due to the stark conceptual differences outlined above, we now cite (Wang et al., 2019) in the context of sleep spindles and memory reactivation (page 15, line 474):

*...Our cross-classification results, indicating that prior learned stimulus categories were decodable during the presence of sleep spindles, are in line with previous work reporting TMR-induced or endogenous memory reactivation locked to spindles (e.g., [48,82–84])...*

Head movements were recorded throughout the wake parts of the experiment (in both groups) using a Polhemus Liberty system. We now assessed whether there would be systematic head-movements during retrieval (-0.5 to 1.5 seconds with regards to the memory prompt onset). Please note that head movement related data during retrieval was available in 18/25 participants in the scalp EEG group and 6/10 patients from the iEEG group. As shown below, the data indicated that the overall head position remained stable and fixed on the center screen (0° head angle) throughout the retrieval period. Non-parametric, cluster-corrected ANOVAs revealed no systematic stimulus-category related differences (scalp EEG group: *p* = 0.16; iEEG group: *p* = 0.13). We added the figure to the Supplemental (Supplementary Figure 24) and refer to it in the main text (page 18, line 588).

*… In case of "old" responses, participants were required to indicate by button press the related head orientation (i.e., the flanking screen on which the image was presented; for head-movement related data during retrieval see Supplementary Fig. 24)…*

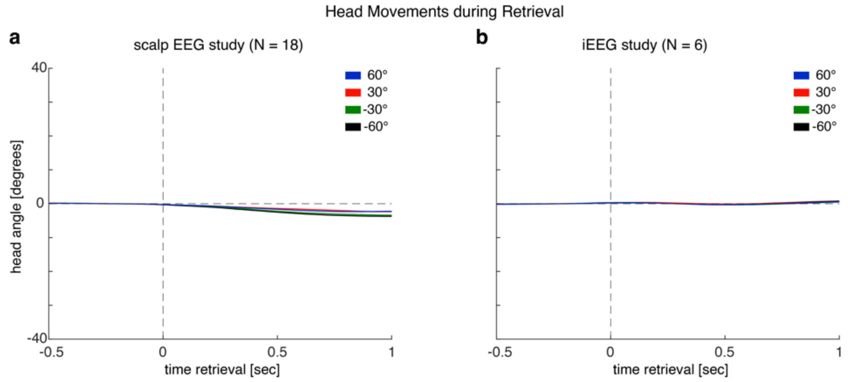


Supplementary Figure 24: Mean horizontal head orientation during associative memory retrieval prior to sleep are depicted. A head angle of 0° corresponds to participants fixating the center screen (during retrieval, participants were instructed to face the center screen, in contrast to the encoding phase, where they moved their gaze to the outer screens). Repeated-measures ANOVAs with 4 levels corresponding to the screen position where the item was presented during encoding (60°, 30°, -30°- -60°) did not show significant differences that survived correction for multiple comparisons during the time period from -0.5 sec to 1 sec (with respect to the onset of the associative memory prompt) in the (a) scalp EEG study (*p*= 0.16, corrected across time) nor in the (b) iEEG study (*p* = 0.13, corrected across time). Head position was available for 18 out of 25 participants in the scalp EEG study and 6 out of 10 patients in the iEEG study.

**Minor concerns:**

Line 51 Were participants given feedback after the pre-sleep test?

Response: Participants did not receive feedback after the pre-sleep test. We added this information (page 4, line 51).

*…Then, for recognized items only, participants indicated which of the four head orientations was associated with the item during the learning phase (associative retrieval, Fig. 1a). Participants received no feedback during the retrieval test. After finishing the memory task, participants went to sleep…*

Line 70: I believe the authors meant to have "cued" here instead of "uncued".

Response: Thanks for spotting this. We corrected the mistake.

Lines 87-9: Great explanation! I was wondering about this while looking at the figure, but this helps explain why there is reactivation before t = 0.

Response: Thank you for the kind feedback.

Line 118: “TMR-induced”

Response: We changed the wording accordingly.

Figure 2: Please define “TFR” somewhere before one encounters it in the figure. It is 'time-frequency representation', right?

Response: We introduce the term TFR (time-frequency representation) now in text (page 6, line 145).

*...To address this question, we correlated, across participants, TMR triggered power as obtained by time-frequency representations (TFR; averaged across the cluster shown in Fig. 2a) and levels of mean classification performance (averaged across the cluster shown in Fig. 2b)...*

Line 209: “TFR-related”

Response: We added a hyphen.

Figure 3: Did I miss something, or is there less of the anticipatory (pre-time 0) reactivation signal here? Is this just random noise or is there a difference in the design somehow?

Response: The Reviewer is correct that the increase in accuracies for the within-retrieval classification of the iEEG study was descriptively delayed as compared to the scalp EEG study (please note that direct comparisons between studies are not possible). The only difference in the experimental design apart from the overall trial number (168 learned images in the scalp EEG study, as compared to 144 in the iEEG study) was that no confidence ratings were obtained in the iEEG study. However, it seems highly unlikely that this difference in the paradigm had an effect on the classification procedure, as confidence ratings in the scalp EEG study were obtained at the end of each trial.
That being said, cross-classification results across studies were highly similar concerning the retrieval-related time-windows that were decodable during TMR, always including pre-memory prompt onset activity (see Figs. 2b, 4e & 5d). In addition, when basing the classification procedure on broadband high-gamma activity in the iEEG study (please see comment 8 by Reviewer 3 and Supplementary Fig. 9), again anticipatory (pre-time 0) reactivation signals became apparent.

Line 285 & Line 345: “instrumental” and “driving” are examples of causal language where correlational language is warranted. Please be mindful of this throughout the paper.

Response: We removed all causal language throughout the manuscript (all changes and deletions are marked in text).

Line 342: many researchers now avoid the word “transfer” with respect to systems consolidation or add nuance that this is likely inexact, incomplete, or memories are instead transformed (such as proposed in Multiple Trace Theory or especially the later Trace Transformation Theory). Please consider changing the wording.

Response: We rephrased the sentence to avoid the concept of “memory transfer” (page 13, line 374).

*…We here used multivariate classification to detect human reactivation processes that are timed by ripples identified in the MTL, providing strong support that ripples in humans initiate memory reactivation akin to animal models [6,16,38]and presumably facilitate the hippocampo-cortical dialogue…*

Line 363-5: Please fix this sentence. I believe they are using “unbeknownst” unusually or inappropriately. I believe the authors mean to say that it is important to consider that, in prior studies showing spindle effects, there could additionally be ripple effects which went undetected due to the lack of appropriate recording method to detect them, but I am not sure.

Response: Thanks for raising the ambiguity in our wording. We rephrased the sentence and do hope that this increased comprehensibility. It now reads as follows (page 14, line 402):

*…Our results indicate that ripples in the MTL peaked during the presence of spindles in trials exhibiting high SO-spindle activity (Fig. 3e), with memory reactivation accompanying those spindle-locked ripples ~~driving memory reactivation~~ (Fig. 4b). This might suggest that also in the previous studies ripples, without being detected due to methodological reasons, were tightly related to ~~driving~~ the decodability of prior learned material during SO-spindles.…*

Line 402: Comma after “memory”

Response: We added a comma.

Line 403: “deteriorated”

Response: We corrected the mistake.

Line 527: was comprised of -> “comprised”

Response: We changed the wording accordingly

**Reviewer #2:**

**Remarks to the Author:**

The study investigated the role of spindle-locked ripples in memory reactivation during human NREM sleep, in two distinct and complementary populations: healthy volunteers (scalp EEG) and patients with implanted electrodes (intracranial EEG). In both cases, participants learned a list of names visually presented at one specific spatial location (one out of five screen monitors horizontally displayed), together with the specific sound associated with each of the four non-central positions. They thus learned both the names to be recognised later amongst distractors, and the association between the name and its spatial location and the associated sound. During the subsequent NREM sleep episode in a TMR procedure, they were presented (cued) with two out of the four sounds (one left- and one right-side association), with the prediction that specific cueing would improve memory performance for the associated material. Unexpectedly, behavioral results showed the opposite pattern, that is performance decreased overnight both for cued and uncued material, but more for the cued than for the uncued material, suggesting an interference effect due to the targeted stimulation. The effect was similar both in healthy volunteers and patients, thus quite robust. At the neural level, MVPA classification during NREM sleep was above chance level to differentiate left- and right-side related cues, as compared to a neutral sound never presented during the learning episode. In healthy subjects, higher levels of SO-spindle activity were associated with increased classification performance, an effect interpreted by the authors as reflecting the promotion of the read-out of TMR-induced memory reactivation. Furthermore, in patients, intracerebral recordings of spindle-locked ripples in the medial temporal lobe highlighted a significant association with classification performance, suggesting that the temporal locking between spindles and ripples within slow association of NREM sleep is instrumental for memory reactivation to unfold (authors' interpretation).

This is an original, very well-designed and written study, with sound methods and analyses, that certainly contributes to our understanding of the associations between spindles, ripples and slow oscillations in the context of information processing. Notwithstanding, I have some reservations about the interpretation of these results in terms of memory reactivation processes, given that all reported effects and associations are obtained with classification performance, which seems to be at least implicitly considered by the authors as a complete surrogate of memory processing. However, they neglect here the fact that behavioural memory performance on the next day was actually worse after cueing than non-cueing, which may cast doubts about the functional relevance of the associations that have been found. In my view, this apparent contradiction needs to be addressed in a more detailed way than it is currently done in the discussion, and claims about the unfolding of memory reactivation processes during NREM sleep in relation with TMR should be toned down in favour a more cautious interpretation. To be clear, I like here very much the work done and do not contest its significance at the neurophysiological level, but have more doubts about the functional interpretation in terms of memory consolidation processes.

Response: We want to thank the Reviewer for their very constructive and encouraging feedback. The Reviewer is right that the effect of TMR on behavior were somewhat surprising. That said, we do not think that the detrimental effect of TMR on behavioral measures contradicts the assumption that TMR-induced memory reactivation during NREM sleep, as picked up by the used classification procedures.

First, TMR did have a robust effect, even though not in the anticipated direction, both across participants and independent studies (scalp and iEEG), indicating that TMR exerted a systematic influence on neural processes during NREM sleep. We would like to point out here that we replicated our behavioral results (and the main EEG results) in an independent sample

(the iEEG group), which is exceptionally rigorous compared to most of the literature in the TMR field.

Second, as pointed out in the discussion (please see below), our results are very much in line with a growing number of findings reporting detrimental effects of TMR on behavior (e.g., Antony et al., 2018; Wang et al., 2019; Witkowski et al., 2021; Joensen et al., 2022). All of these studies, including ours, have one feature in common: several targets were associated with one memory cue, which led to the suggestion that if associations between multiple targets and one cue vary in strength, TMR might elicit the reactivation of targets most strongly associated with the cue (Joensen et al., 2022). Importantly, our result indicating that our decoding results have been driven by high confidence trials (see Supplementary Fig. 23) are very much in line with this hypothesis. Third, multivariate analysis methods (such as MVPA or representational similarity analysis) have been proven valuable tools to identify memory reactivation during both wakefulness and sleep (Lewis and Bendor, 2019; Schreiner and Staudigl, 2020). Our classification results in both the scalp EEG and the iEEG study, indicating that prior learned stimulus categories were decodable during the presence of sleep spindles, are very much in line with previous work showing TMR-induced but also endogenous memory reactivation during related time windows (i.e., connected to the presence of sleep spindles), using similar classification procedures (e.g., Cairney et al., 2018; Schreiner et al., 2021; Ngo and Staresina, 2022). This consistency across independent studies, especially in the case of studies were TMR exhibited beneficial effects on behavior (Cairney et al., 2018; Ngo and Staresina, 2022), provides further indication that our TMR procedure induced memory reactivation but with an opposite consequence on behavior. Finally, the obtained cross-classification results were highly specific with regards to retrieval processes. Retrieval-related neural patterns of head-orientations could only be successfully classified during TMR in case of correctly remembered head-orientations (see Supplementary Fig. 5 for cross-classification based on not-remembered trials and below), while decodable time windows with regards to the retrieval data highly overlapped between the cross-classification and within retrieval classification procedures.

That said, we reviewed the whole manuscript for claims relating the outcomes of the classification results to memory consolidation, removed those interpretations and in general all causal language (all changes and deletions are marked in text).

In addition, we added a section to the discussion, summarizing the points above (page 15; line 466):

*… We here used TMR as an experimental tool to trigger memory reactivation during sleep. It has been shown that TMR modulates memory, leading most often to performance increases [28,62–66]. Hence, it might seem surprising that TMR did not benefit but deteriorated memory performance both in healthy participants as well as in patients. However, a growing number of studies report TMR-induced impairments, in particular when several targets were associated with a given TMR cue[30,67,68]. In the present study, multiple images (42 in the scalp EEG study; 36 in the intracranial EEG study) were associated with each of the four head orientations. It has been suggested that if the associations between multiple targets and one cue vary in strength, TMR might elicit the reactivation of targets most strongly associated with the cue[67], akin to models describing retrieval competition during wake [69,70]. Selectively strengthening a subset of strong cue-target associations via TMR, however, might lead to weakly associated targets losing the competition for being reactivated during a subsequent memory test. Depending on the relative amount of cue-target associations in either subset, this might show as a net beneficial, detrimental, or no effect of TMR on memory performance. Interestingly, in an exploratory analysis (see Supplementary Fig. 12), we found a positive correlation between memory confidence in the pre-sleep memory test and the effect of TMR. Assuming that confidence ratings in our study are positively related to memory strength [71,72], but see [73], this relationship indicates that TMR may have mainly reactivated targets that were strongly associated with their cues and further strengthened their association. In turn, these strong targets might have outcompeted weaker ones*

*when competing for being retrieved during post-sleep retrieval, resulting in the observed detrimental effect of TMR on memory performance.*

*In sum, several lines of argument support the assumption that our classification procedure indeed picked up neural signatures of memory reactivation, despite the fact that TMR exerted a detrimental influence on memory performance. First, TMR did have a robust effect on behavior, both across participants and independent studies (scalp and iEEG), indicating a systematic influence on neural processes during NREM sleep. Second, as outlined in detail above, a growing number of studies reported similar detrimental effects on behavior when several targets were associated with a given TMR cue [30,74,75,81]. Third, multivariate analysis methods (such as MVPA [86] or representational similarity analysis [87]) have been proven valuable tools to identify memory reactivation during both wakefulness and sleep [5,77]. Our cross-classification results, indicating that prior learned stimulus categories were decodable during the presence of sleep spindles, are in line with previous work reporting TMR-induced or endogenous memory reactivation locked to spindles (e.g., [48,82–84]). This consistency with other studies, especially those where TMR exhibited beneficial effects on behavior [83,84], provides further indication that our TMR procedure indeed induced memory reactivation, but with a reversed consequence on behavior. Finally, the obtained cross-classification results were highly specific with regards to retrieval processes. Retrieval-related neural patterns of head-orientations could only be successfully classified during TMR in case of correctly remembered head-orientations (see Supplementary Fig. 5 for cross-classification based on not-remembered trials), while decodable time windows with regards to the retrieval data highly overlapped between the cross-classification and within retrieval classification procedures…*

Page 6, line 138:

*…(associative retrieval time-window: -110 to 330 ms; the fact that decodability preceded the onset of the associative memory prompt again indicates that associative retrieval processes were probably ignited during the preceding recognition memory test; for cross-classification based on not-remembered retrieval trials see Supplementary Fig. 5)…*

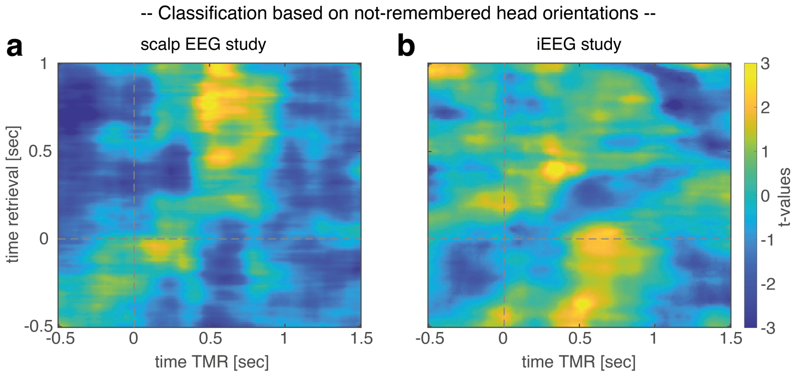


Supplementary Figure 5: Not-remembered head-orientations (left vs. right) were (a) neither decodable during TMR in the scalp EEG study ($p = 0.14$), (b) nor in the iEEG study ($p = 0.88$).

**Besides this major comment, here below other remarks:**

1. the way the paper is written suggests that memory consolidation would take place only during sleep, which is exaggerated. E.g., see the first line of the abstract, at least it should be said that memory consolidation PARTIALLY relies on the reactivation of previous experience during sleep; or the first line in the introduction which is also ambiguous in this respect.

<u>Response:</u> We now make clear that memory consolidation only in part relies in sleep-related processes such as reactivation.

Abstract: *Memory consolidation relies in part on the reactivation of previous experiences during sleep…*

Introduction (page 3, line 3): *…Contemporary models propose that memory consolidation, i.e., the strengthening of memories during sleep, is achieved in part by reactivating experiences that were encoded during prior wakefulness* [1,2]*...*

2. page 4, behavioural results: please clarify how memory performance is computed. Based on the claim in the last sentence that there is no effect for recognition memory, I assume that it is the association between the name and its location, but it needs to be better clarified. Also, in the sentence lines 69-70, it is said that uncued items are better remembered than uncued items. Probably a typo.

Response: The memory tests (both pre- and post-sleep) included the 168 images from encoding (old items) intermixed with 84 new images, which were not seen by the participants before (please note in the iEEG study the stimulus material consisted of 144 old items and 72 new items). Each trial started with a fixation cross, after which one of the images was presented on the center screen. Subsequently, recognition memory was assessed. Participants were asked to indicate whether the presented image was "old" (i.e., part of the learning material) or "new"' (i.e., it was not seen during learning). In case of "new" responses, participants immediately moved on to the next trial. In case of "old" responses, participants were required to indicate the related head orientation by button press, which corresponds to the associative memory retrieval. The impact of TMR on recognition memory was assessed via the hit rate (correctly recognized old items). We tried to make this rationale now clearer both in the main text (page 4, line 71) and the methods section (page 18, line 583).

Main text:

*…For recognition memory (hit rate from old/new judgments), we neither found a significant main effect of test time ($F_{1,24} = 0.29$; $p = 0.59$); nor a significant interaction between test-time and cueing ($F_{1,24} = 0.08$; $p = 0.77$; see Supplementary Fig. 1 for details)...*

Methods:

*…Each trial started with a fixation cross, presented for 1.5 ± 0.1 s. After the fixation cross, an image was presented on the center screen. After 1 second, recognition memory was assessed. Participants were asked to indicate whether the image was "old" (i.e., part of the learning material) or "new"' (i.e., it was not seen during learning) within the next 10 s…*

We corrected the typo indicated by the Reviewer (page 4, line 70):

*…Follow up post-hoc t-test (relative memory performance pre- to post-sleep) also indicated that uncued items were better remembered as compared to ~~un~~cued items ($t_{1,24} = 2.747$; $p = 0.011$)…*

3. Lines 78-8: could you explain why pulling together bot pre- and post-sleep session to extract head-orientation related neuronal activity during retrieval? Would it not be more appropriate to use only pre-sleep activity as it is expected that post-sleep activity would be modulated by the night TMR manipulation?

Response: The reason for pulling together data from both the pre- and post-sleep retrieval data was to increase the number of trials for the decoding procedure (both for the within retrieval decoding, but likewise for the cross-classification), as classification robustness and

fidelity heavily depends on trial numbers. In addition, only those items that were remembered in both retrievals were taken into account in all classification related analyses, rendering the pre- and post-sleep retrieval data comparable. That said, we repeated the within-retrieval classification for both the scalp and the intracranial EEG groups. As shown below cutting trial numbers into half by only using the pre-sleep retrieval data impacted the robustness of the results. The general temporal evolution of the obtained accuracies followed our original results (with accuracies ascending in an early time-window after the onset of the memory prompt). However, while significant above chance classification emerged in the scalp EEG data ($p$ = 0.043, time-window: 190 to 300ms), no significant time-window was observable for the iEEG data ($p > 0.05$), further highlighting the advantage of high trial numbers for classification and hence our approach of pooling the retrieval data.

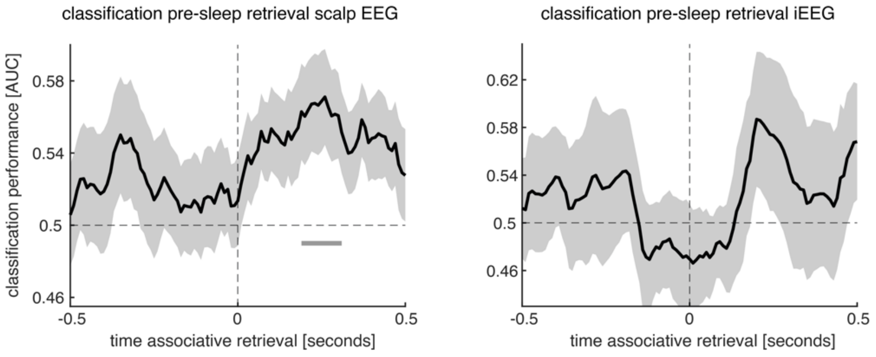


4. Learning task: it is said that the screens are positioned at - or + 30° and - or + 60°, does that correspond to the visual angle? What was the screen to eye distance and the size of the monitors?

Response: Yes, the positions of the flanking monitors (-60°, -30°, 30°, 60°) corresponds to the visual angle with respect to the participants. But please note that changes in the sitting position, head orientation etc. might have led to variance concerning the exact visual angles across participants. The screen to eye distance was ~1 meter for the scalp EEG study and ~1.20 meter for the iEEG study. In both studies a 28 inch monitor was used as central monitor, while the flanking monitors were 22 inch each.

We added this information to the Methods section (page 17, line 555):

*…Participants / patients began by fixating on the center screen (screen size: 28 inch), where a fixation cross was presented for 1.5 ± 0.1 seconds. The screen to eye distance was ~1 meter for the scalp EEG study and ~1.20 meter for the iEEG study. The cross disappeared and one of four animal sounds was played (600 ms). Subsequently, the cross appeared on one of four flanking screens (positioned at -60°, -30°, +30° and 60° relative to the center screen, duration: 2.5 seconds, see Fig. 1a; size of flanker screens: 22 inch)…*

5. As mentioned above, assuming that cued-related MVPA classification performance is equivalent to memory processing performance is an analogical assumption; this should be better discussed and grounded by the authors.

Response: As outlined above (response to first major comment), we reviewed the whole manuscript for claims relating the outcomes of the classification results to memory consolidation, removed those interpretations and in general all causal language (all changes and deletions are marked in text) and added a dedicated section to the discussion concerning the relationship of multivariate classification and memory reactivation.

6. It is said (e.g., lines 218-219) that SO-spindle activity coordinates temporal occurrence of ripples, but the association is with TFRs that do not have high temporal resolution. Would it be possible for instance to try to time-lock the beginning of the ripples with the beginning of the spindles?

Response: To assess the temporal relationship between MTL ripples and cortical spindles, we created peri-event time histograms (bin size = 50 ms) where cortical spindle onsets served as seed (time = 0), while the targets (MTL ripple onsets) are depicted relative to the seed (i.e., spindle onsets). The resulting histogram was normalized by the total number of detected spindles (multiplied by 100) per participant. As with the Modulation Index (Fig. 5c), cortical contacts exhibiting the strongest power in the spindle band (12-15 Hz; 0 – 1.5 seconds after cues onset) provided the data for this analysis. As shown below (and in line with our TRF related results) ripples tended to ignite towards the center of sleep spindles (the solid horizontal line indicates the mean spindle duration (mean spindle duration: 0.75 ± 0.008 sec; peak latency: 0.37 ± 0.042 sec in relation to spindle onsets). We added this result to Fig. 5c and refer to it in text (page 11, line 317).

*…When assessing the preferred phase of spindles for their grouping of ripples, we found that ripples were nested towards the trough of cortical spindles (Fig. 5c top inset; V-test against ± pi: V = 5.29, p = 0.022; mean coupling direction: -176.67 ± 16.61°; mean vector length = 0.21± 0.031)[17,21], while ripples generally tended to emerge towards spindle centers (see Fig. 5c bottom inset)…*

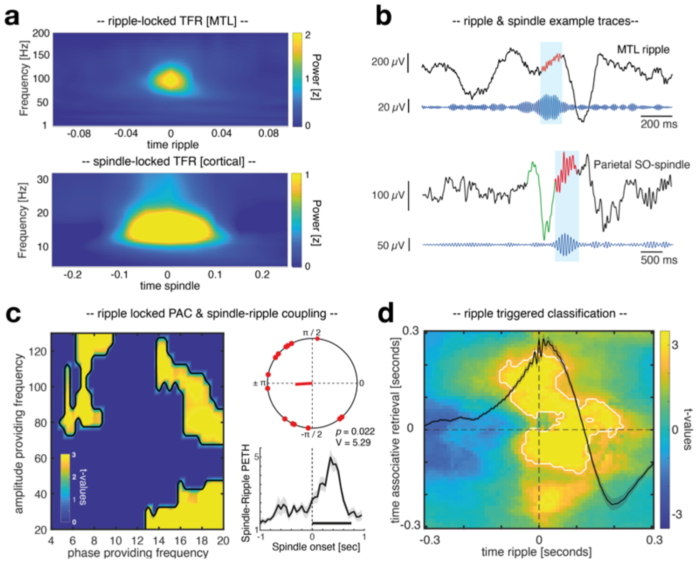


Fig.5 Spindle-ripple interactions and ripple-locked classification. (a) Time-frequency-representations of ripple-locked MTL data segments (top) and spindle-locked data segments from frontal, parietal and temporal contacts (bottom). (b, top) Unfiltered iEEG trace from single contact in the MTL with ripple band filtered signal (80 to 120 Hz) shown in blue. Blue shaded area highlights a representative ripple. (b, bottom) Unfiltered iEEG trace from single parietal contact with spindle band filtered signal (12 to 15 Hz) shown in blue. Blue shaded area highlights a representative spindle following a SO (green). (c) Assessing phase-amplitude coupling (PAC) using the Modulation Index revealed that the phase of cortical spindles influenced amplitudes in the ripple range in MTL contacts (~ 80 – 120 Hz; *p* = 0.005, corrected). In addition, cortical delta/theta phase exhibited a significant effect on MTL ripple amplitudes (*p* = 0.007, corrected), while the spindle phase additionally modulated low gamma amplitudes in the MTL (~20 – 40 Hz; *p* < 0.001, corrected). The top inset illustrates phases of the spindle-ripple modulation, indicating a clustering of ripples towards spindle troughs (corresponding to ±π; V test against ±pi: v = 5.29, p = 0.022; mean coupling direction: -176.67 ± 16.61°, mean vector length = 0.21± 0.031). The bottom inset illustrates the temporal modulation of MTL ripple onsets by cortical spindle onsets (ripple percentage in relation to spindles ± 1 sec; mean ± SEM across MTL contacts). Solid horizontal line indicates mean spindle duration (mean spindle duration: 0.75 ± 0.008 sec; peak latency: 0.37 ± 0.042 sec in relation to spindle onsets). (d) Head orientation-related brain patterns (left vs. right) were decodable during the presence of spindle-locked MTL ripples (contour lines indicate the extent of the significant cluster, *p* = 0.007 corrected; color range (blue to yellow) represents t values).

**Reviewer #3:**
**Remarks to the Author:**

The study investigates the relationship between memory reactivation during sleep and the occurrence of ripple-spindle complexes, using the Targeted Memory Reactivation (TMR) paradigm. Despite a slight deterioration in memory performance post-TMR, the results effectively elucidate the association between memory reactivation and the timing of ripple/spindle complexes during sleep and suggest an interesting link between confidence rating and the TMR effect. This is a novel contribution that significantly advances the field and will be of interest to a large audience. Overall, the paper is well-written, and the results are conveyed clearly and effectively. Nevertheless, to make the results more compelling and to rule out alternative explanations, the authors need to address several critical issues and conduct additional analysis, as detailed below:

## Major points

1) While the authors do illustrate a significant enhancement in the slow oscillation (SO) and spindle power, it would be valuable to include raw spectrograms that visibly depict spindle activity during the different TMR conditions. Merely showcasing power increases represents just one facet of the analysis; it is crucial to consider additional spindle characteristics as well (duration, spatial distribution, and mean frequency).

Response: Following the Reviewers suggestion we now included raw spectrograms of the learning-related and the control TMR conditions. We used electrode Cz as exemplary EEG electrode for the scalp EEG study and all available frontal contacts (N = 156) for the iEEG study. In line with Fig. 2a, activity in the sleep spindle band was clearly stronger following experimental cues as compared to control cues in both groups. We added the figure to the Supplemental (Supplementary Figure 13) and refer to it in the main text (page 9, line 242).

*…(low frequency cluster: p < 0.001; spindle cluster: p < 0.001; corrected for multiple comparisons across time and frequency, Fig. 4c; for raw spectrograms of different TMR conditions see Supplementary Fig. 9)…*

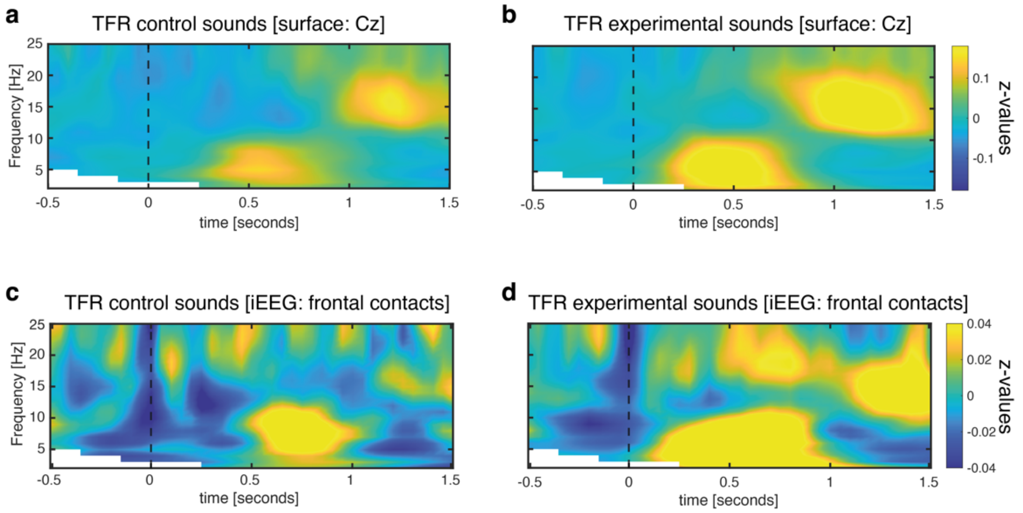


Supplementary Figure 13: (a + b) Average time–frequency representations for control sounds (a) and experimental sounds at electrode Cz (b) for the scalp EEG study. (c + d) Time–frequency representations for control sounds (c) and experimental sounds averaged across all frontal contacts (N = 156) (d) for the iEEG study.

Furthermore, we now report additional spindle characteristics in both experimental groups (scalp EEG and iEEG). Specifically, we assessed potential differences in spindle duration,

spindle frequency, relative spindle number and the timing of spindles after cue onset (i.e., spindle density) between experimental and the control sound condition.

As shown below, neither spindle duration nor spindle frequency differed significantly between conditions in the scalp EEG study (mean duration experimental sounds: 0.7403 ± 0.0013 seconds, mean duration control sounds: 0.7439 ± 0.0019, $p$ = 0.9; mean frequency experimental sounds: 14.03 ± 0.008, mean frequency control sounds: 14.01 ± 0.009, $p$ = 0.12). In contrast the relative number of spindles (spindles emerging after cue onset in a time window between 0 and 1.5 seconds, normalized by the number of trials per condition), differed significantly between conditions. Experimental sounds were associated with more sleep spindles across frontal, central and parietal areas as compared to control sounds (relative number experimental sounds: 0.32 ± 0.018, relative number control sounds: 0.30 ± 0.019, $p$ = 0.002). Finally, we assessed whether the timing of spindle emergence would differ between conditions. We created for each condition peri-event histograms (bin size = 50 ms) of spindle events time-locked to TMR cues (experimental or control sounds, respectively). The resulting histograms were normalized by the number of trials per condition. We found that the higher number of spindles, as reported above, was attributable to a time-window between 0.7 and 1.25 seconds after cue onset ($p$ < 0.001). We added the figure to the Supplemental (Supplementary Figure 4) and refer to it in the main text (page 6, line 124).

*…Consistent with previous findings [29–31], learned TMR cues, as compared to control cues, triggered a significant power increase in the SO-spindle range (i.e., an initial low frequency burst followed by a fast spindle burst; p <0.001, corrected for multiple comparisons across time, frequency, and space; see Fig. 2a), foreshadowing that learning-related TMR cues might have triggered relevant neuronal processing in the sleeping brain (for spindle characteristics associated with experimental and control sounds see Supplementary Fig. 4)…*

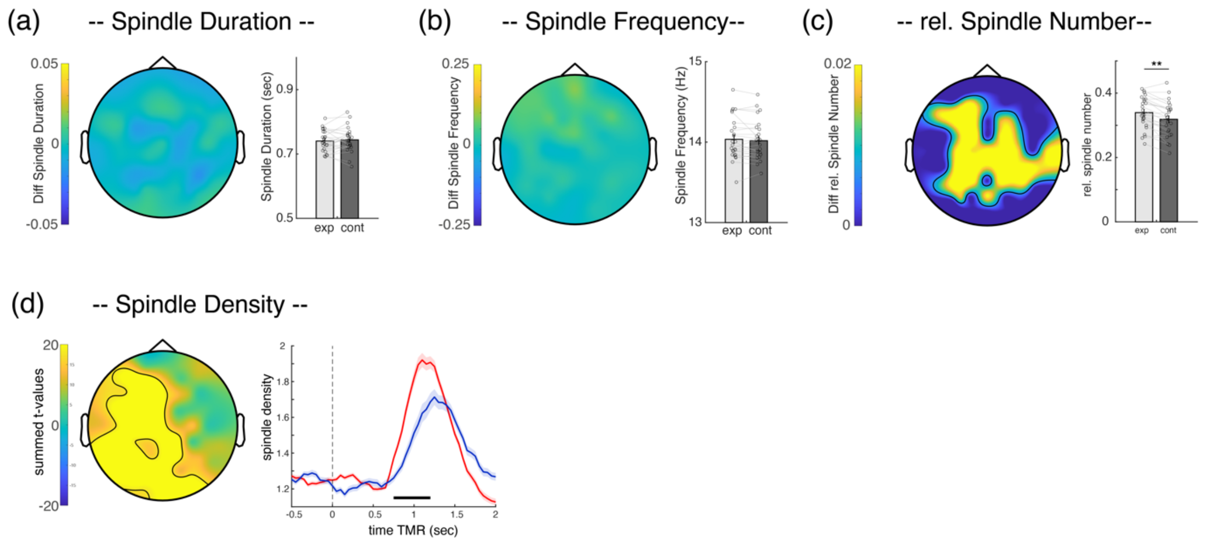


Supplementary Figure 4: (a) Spindle duration did not differ between conditions (experimental vs. control sounds) in the scalp EEG study (mean spindle duration experimental sounds: 0.7403 ± 0.0013 seconds, mean spindle duration control sounds: 0.7439 ± 0.0019, $p$ = 0.9, corrected across electrodes; the bars represent the mean spindle duration across all electrodes). (b) Spindle frequency did not differ between conditions (mean spindle frequency experimental sounds: 14.03 ± 0.008, mean spindle frequency control sounds: 14.01 ± 0.009, $p$ = 0.12, corrected across electrodes; the bars represent the mean spindle frequency across all electrodes). (c) The relative number of spindles (spindles emerging after cue onset in a time window between 0 and 1.5 seconds, normalized by the number of trials per condition), differed significantly between conditions. Experimental sounds were associated with more sleep spindles across frontal, central and parietal areas as compared to control sounds (relative spindle number experimental sounds: 0.32 ± 0.018, relative spindle number control sounds: 0.30 ± 0.019, $p$ = 0.002, corrected across electrodes; the bars represent the mean relative spindle number across significant electrodes). (d) Comparing the occurrence probabilities of experimental sound (red) and control sound (blue) related spindles revealed higher spindle numbers for experimental sounds between 0.7 and 1.25 seconds after cue onset ($p$ < 0.001, corrected for multiple comparisons across time points and electrodes).

For the iEEG study we focused our analyses on frontal, parietal and temporal contacts (N = 317) as regions of interest for spindle emergence and delineating their characteristics. Also here, we did not find any differences between conditions related to spindle duration or spindle frequency (mean spindle duration experimental sounds: 0.709 ± 0.0032 seconds, mean spindle duration control sounds: 0.711 ± 0.0037, $p$ = 0.55; mean spindle frequency experimental sounds: 14.38 ± 0.02, mean spindle frequency control sounds: 14.35 ± 0.01, $p$ = 0.07).

Again the relative number of spindles (spindles emerging after cue onset in a time window between 0 and 1.5 seconds, normalized by the number of trials per condition), differed significantly between conditions across all contacts (relative spindle number experimental sounds: 0.201 ± 0.0033, relative spindle number control sounds: 0.181 ± 0.0034, $p < 0.001$). To assess whether relative spindle numbers would differ across regions, we repeated the analysis specifically taking only frontal (N = 156), parietal (N = 46) or temporal contacts (N = 115) into account. We found that the relative number of spindles following experimental sounds was higher in any of the regions as compared to control sounds (frontal: relative spindle number experimental sounds: 0.196 ± 0.0048, relative spindle number control sounds: 0.179 ± 0.0047, $p < 0.001$; parietal: relative spindle number experimental sounds: 0.244 ± 0.0085, relative spindle number control sounds: 0.208 ± 0.0084, $p < 0.001$; temporal: relative spindle number experimental sounds: 0.189 ± 0.0049, relative number control sounds: 0.173 ± 0.0043, $p < 0.001$). A linear regression further indicated that the difference in the relative spindle number was not reliably influenced by the region of detection (F = 0.21, $p$ = 0.64).

Finally we assessed across frontal, parietal and temporal contacts whether the timing of spindle emergence would differ between conditions using peri-event histograms. We found that the higher number of spindles, as reported above, was attributable to a time-window between 0.85 and 1.1 seconds after cue onset ($p$ = 0.027), very much in line with the scalp EEG study. Again, we tested whether this effect would vary across conditions. In all regions, occurrence probabilities of experimental sound related spindles were higher around 1 second post-stimulus, indicating again no clear difference in spindle emergence across areas (frontal: 0.8 – 1.5 seconds, $p < 0.001$; parietal: 0.65 – 1.05 seconds, $p < 0.001$; temporal: 0.65 – 1.05 seconds, $p$ = 0.03). These findings replicate findings in the scalp EEG study, where higher number of spindles were found across frontal, parietal and temporal areas for experimental as compared to control cues.

We added the figure to the Supplemental (Supplementary Figure 14) and refer to it in the main text (page 9, line 243).

*…(low frequency cluster: p < 0.001; spindle cluster: p < 0.001; corrected for multiple comparisons across time and frequency, Fig. 4c; for raw spectrograms of different TMR conditions see Supplementary Fig. 13; for spindle characteristics associated with experimental and control sounds see Supplementary Fig. 14)…*

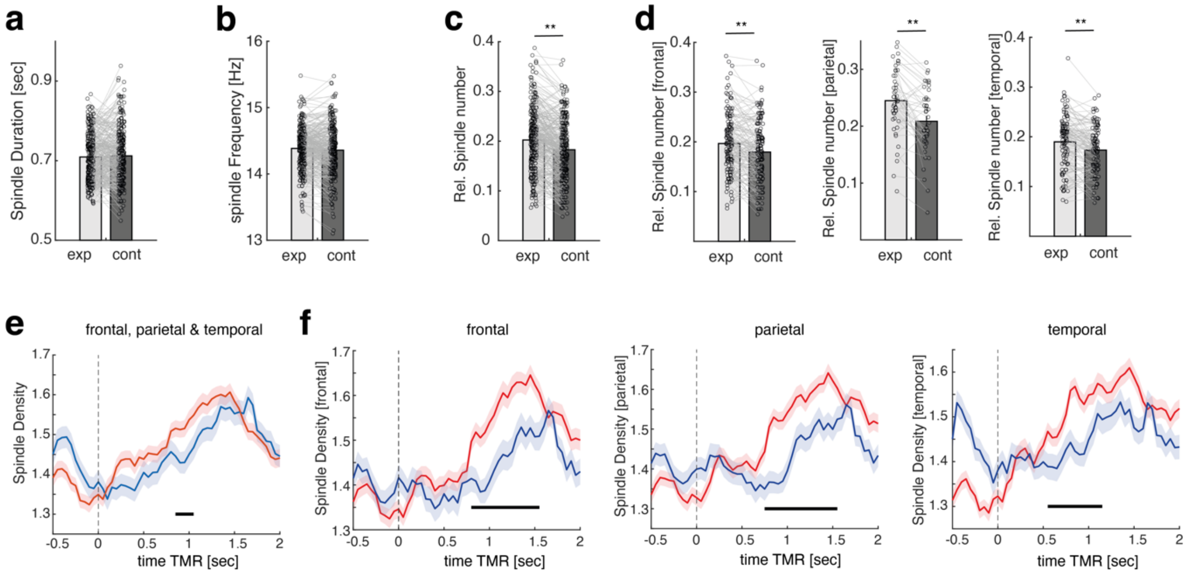


Supplementary Figure. 14: (a) Spindle duration did not differ between conditions (experimental vs. control sounds) in frontal, parietal and temporal intracranial contacts (mean spindle duration experimental sounds: 0.709 ± 0.0032 seconds, mean spindle duration control sounds: 0.711 ± 0.0037, $t_{1,316}$ = -0.59, $p$ = 0.55; the bars represent the mean spindle duration across all contacts (N = 317)). (b) Spindle frequency did not differ between conditions (mean spindle frequency experimental sounds: 14.38 ± 0.02, mean spindle frequency control sounds: 14.35 ± 0.01, $t_{1,316}$ = 1.77, $p$ = 0.07). (c) The relative number of spindles (spindles emerging after cue onset in a time window between 0 and 1.5 seconds, normalized by the number of trials per condition), differed significantly between conditions. Experimental sounds were associated with more sleep spindles as compared to control sounds (relative spindle number experimental sounds: 0.201 ± 0.0033, relative spindle number control sounds: 0.181 ± 0.0034, $t_{1,316}$ = 8.16, p < 0.001). (d) This effect became apparent in all examined regions (frontal contacts (N = 156): relative spindle number experimental sounds: 0.196 ± 0.0048, relative spindle number control sounds: 0.179 ± 0.0047, $t_{1,155}$ = 4.78, $p$ < 0.001; parietal contacts (N = 46): relative spindle number experimental sounds: 0.244 ± 0.0085, relative spindle number control sounds: 0.208 ± 0.0084, $t_{1,45}$ = 4.83, $p$ < 0.001; temporal contacts (N = 115): relative spindle number experimental sounds: 0.189 ± 0.0049, relative spindle number control sounds: 0.173 ± 0.0043, $t_{1,114}$ = 5.03, p < 0.001). (e) Comparing the occurrence probabilities of experimental- (red) and control sound (blue) related spindles, revealed higher spindle numbers for experimental sounds between 0.85 and 1.1 seconds after cue onset across all frontal, parietal and temporal contacts ($p$ = 0.027, corrected across time). (f) Occurrence probabilities of experimental sound related spindles were higher around 1 second post-stimulus in all examined regions (frontal: 0.8 – 1.5 seconds, $p$ < 0.001; parietal: 0.45 – 1.05 seconds, $p$ < 0.001; temporal: 0.65 – 1.05 seconds, $p$ = 0.03; all corrected across time).

2) Is there a difference in the spatial distribution of spindles between experimental and control sounds in the iEEG data? such analysis will strengthen the argument for functionally distinct ripples/spindles induced by the TMR cues.

Response: Please see our response to the Reviewer's point 1, which shows no differences in spatial distribution of spindles in the iEEG data between experimental and control sounds (e.g., no significant effect of region in linear regression analysis; F = 0.21, $p$ = 0.64). For the reviewer's convenience, we plot a spatial distribution of the difference in the relative number of spindles during TMR trials (experimental minus control cues, 0 – 1.5 seconds after cue onset) for all frontal, parietal and temporal contacts. In line with the results reported above, no clear topographical hotspot for experimental cue induced spindles as compared to control cues became apparent (please see below). Given that the number of supplementary figures is already very high, we did not add this figure to the supplements, since it adds no critical information.

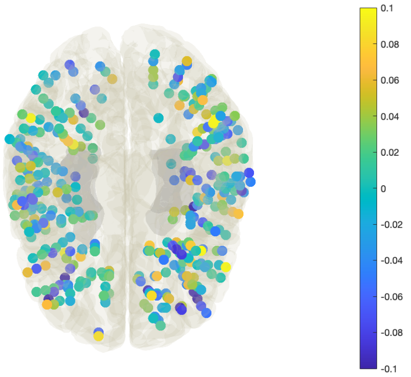


3) The authors should include figures showing the spectrotemporal signature of ripples and spindles in their data, as well as 2-3 example traces. As detailed in Liu et al (https://www.nature.com/articles/s41467-022-33536-x) the authors should report ripple rate across the different sleep stages, to demonstrate what we already know about ripples, namely, that ripple rate is generally higher during non-REM sleep compared to REM. This will further convince the readers that the authors detected genuine physiological ripples.

Response: Following the Reviewers suggestion we detected ripples in the MTL during both NREM and REM sleep. In line with previous findings in humans (Chen et al., 2021), ripple density during NREM sleep was generally higher as compared to REM sleep (NREM: 0.19 ± 0.017 ripples per second (overall N = 3214 ± 393); REM: 0.09 ± 0.011 ripples per second (overall N = 523 ± 54.18); $t_{1,14}$ = 4.81, $p < 0.001$). We added the figure to the Supplemental (Supplementary Figure 11) and refer to it in the main text (page 9, line 248).

*…Ripples were extracted in MTL contacts (7 patients, 14 contacts; see Methods for details; see Fig. 4a and b; see Supplementary Fig. 11 for a comparison of ripple rates between NREM and REM sleep [38])…*

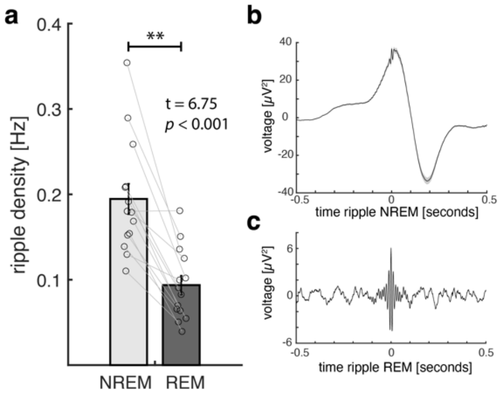


Supplementary Figure 15: (a) Ripple density during NREM sleep was higher as compared to REM sleep (NREM: 0.19 ± 0.017 ripples per second; REM: 0.09 ± 0.011 ripples per second; $t_{1,14}$ = 4.81, $p < 0.001$). (b) Ripple-centered grand average of all detected ripples during NREM sleep, locked to maximal negative amplitude (N = 3214 ± 393). (c) Ripple-centered grand average of all detected ripples during REM sleep, locked to maximal negative amplitude (N = 523 ± 54.18).

In addition, we performed time-frequency analyses of (i) ripple-locked data segments from MTL contacts (N = 14) and (ii) spindle-locked data segments from frontal, parietal and temporal contacts (N = 317). Both ripples and spindles were extracted between 0 and 1.5 seconds with regards to the cue onset. We incorporated the time-frequency representations as well as

example traces for ripples and spindles into Figure 5 and refer to them in text (page 11, line 307).

*…Having established that cardinal sleep oscillations and reactivation of head orientation-related activity co-occur in time, we next assessed whether ripples and their coupling to spindles would be essential for triggering reactivation processes (for time-frequency representations and single trial examples of spindles and ripples see Fig. 5a + b)…*

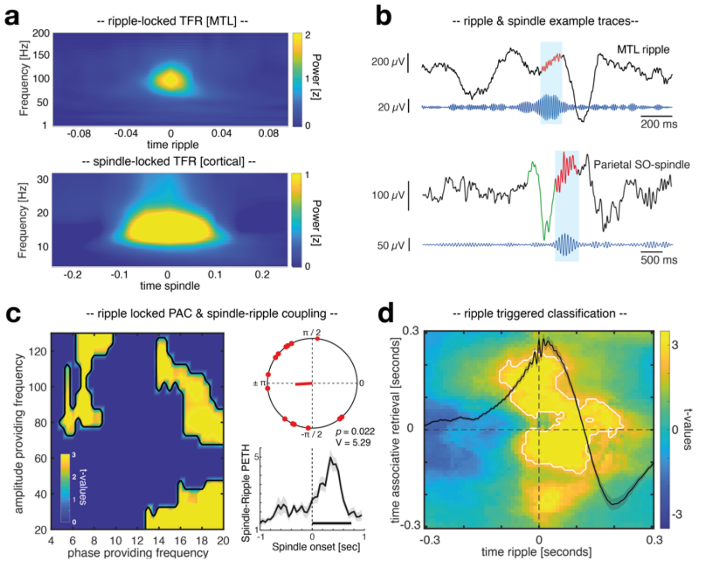


Fig.5 Spindle-ripple interactions and ripple-locked classification. (a) Time-frequency-representations of ripple-locked MTL data segments (top) and spindle-locked data segments from frontal, parietal and temporal contacts (bottom). (b, top) Unfiltered iEEG trace from single contact in the MTL with ripple band filtered signal (80 to 120 Hz) shown in blue. Blue shaded area highlights a representative ripple. (b, bottom) Unfiltered iEEG trace from single parietal contact with spindle band filtered signal (12 to 15 Hz) shown in blue. Blue shaded area highlights a representative spindle following a SO (green). (c) Assessing phase-amplitude coupling (PAC) using the Modulation Index revealed that the phase of cortical spindles influenced amplitudes in the ripple range in MTL contacts (~ 80 – 120 Hz; $p$ = 0.005, corrected). In addition, cortical delta/theta phase exhibited a significant effect on MTL ripple amplitudes ($p$ = 0.007, corrected), while the spindle phase additionally modulated low gamma amplitudes in the MTL (~20 – 40 Hz; $p < 0.001$, corrected). The top inset illustrates phases of the spindle-ripple modulation, indicating a clustering of ripples towards spindle troughs (corresponding to ±π; V test against ±pi: v = 5.29, p = 0.022; mean coupling direction: -176.67 ± 16.61°, mean vector length = 0.21± 0.031). The bottom inset illustrates the temporal modulation of MTL ripple onsets by cortical spindle onsets (ripple percentage in relation to spindles ± 1 sec; mean ± SEM across MTL contacts). Solid horizontal line indicates mean spindle duration (mean spindle duration: 0.75 ± 0.008 sec; peak latency: 0.37 ± 0.042 sec in relation to spindle onsets). (d) Head orientation-related brain patterns (left vs. right) were decodable during the presence of spindle-locked MTL ripples (contour lines indicate the extent of the significant cluster, $p$ = 0.007 corrected; color range (blue to yellow) represents t values).

4) To facilitate comparison with analogous studies examining awake human ripples (Vaz et al., 2019; Norman et al., 2019, 2021; Henin et al., 2021; Dickey et al., 2022; Kunz et al., 2023;), the authors should present the data in Fig 3e and Supplementary Figure 8 in terms of ripple rate (events/sec), and include a single trial visualization of ripples relative to the retrieval and TMR cues (either ripple raster or ripple density showing pooled-trials time).

<u>Response:</u> In order to improve comparability with similar studies examining human ripples during wakefulness, we exchanged Fig 3e and Supplementary Fig 8 and now illustrate the ripple rate in Hz.

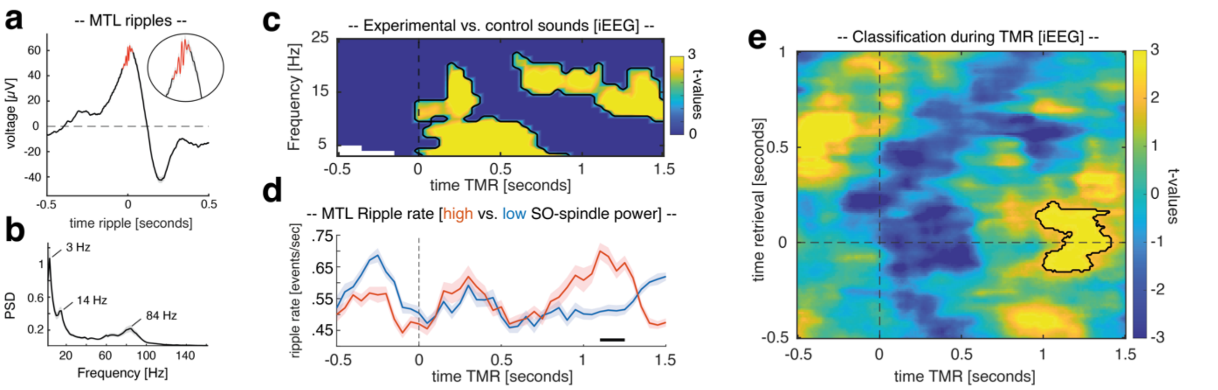


Fig.4. intracranial EEG study: TMR (a) Ripple-triggered grand average of all detected ripples (7 patients, 14 contacts; locked to maximal negative amplitude) during TMR (-.5 to 1.5 seconds; 138.78 ± 21.72 ripples in 231.14 ± 19.94 trials). A zoomed version of the ripples is illustrated in the lower inset. (b) Power spectral density (PSD) averaged across all detected SWRs [± 300 ms] indicating distinct peaks in the SO/delta, spindle and ripple range (i.e., 3 Hz, 14 Hz and 84 Hz). (c) Power difference indicates that retrieval-related TMR cues triggered increased power in intracranial EEG recordings ($p < 0.05$, corrected) as compared to control cues. (d) Ripple rates (events per second) for trials exhibiting high (red) and low power (blue) in the SO-spindle range, respectively. Ripple rates differed significantly between conditions ($p = 0.027$; corrected), with MTL ripples peaking during elevated spindle activity. (e) Head orientation-related brain patterns (left vs. right) were decodable during TMR when contrasting high and low SO-spindle activity trials (contour lines indicate the extent of the cluster, $p = 0.019$ corrected; color range (blue to yellow) represents t values against chance level performance.

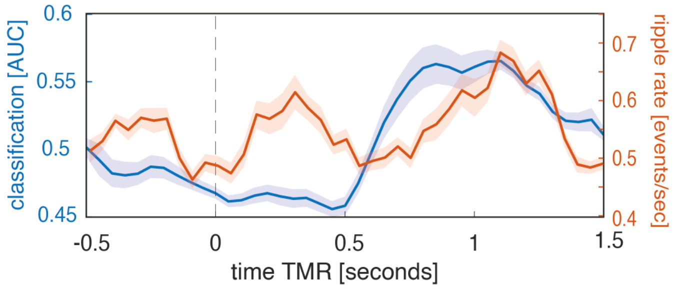


Supplementary Figure 17. Time course of ripple rate (events / sec) and reactivation signal. Blue: Classification output averaged across the relevant retrieval time [-150 to 200 ms]. Red: Patient-averaged ripple rate across time.

In addition, we added exemplary raster plots for TMR ripples during high and low SO-spindle power trials as well as for retrieval-related ripples during remembered and not-remembered trials to the Supplemental (Supplementary Figures 11 and 18) and refer to it in text.

Page 8, line 201:

*…(see Methods for details; see Fig. 3c and 3d for ripples related EPRs and time-frequency representations; see Supplementary Fig. 11 for exemplary raster plots of retrieval-related ripples during remembered and not-remembered trials)…*

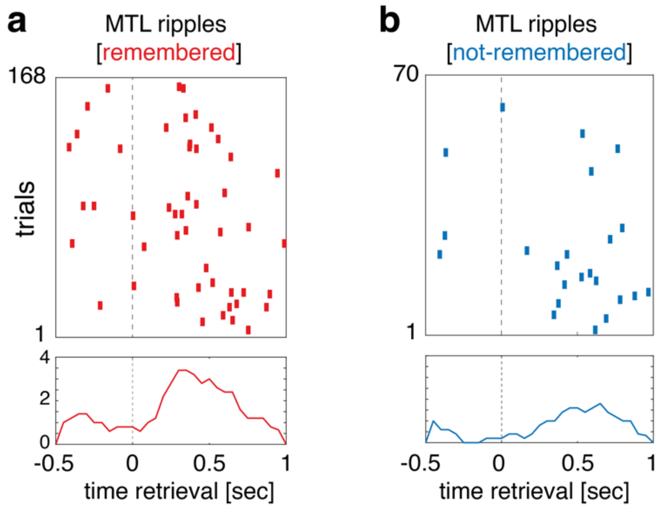


Supplementary Figure 11. (a) Exemplary raster plots for retrieval-related MTL ripples during remembered (red) and (b) not-remembered trials. Bottom inserts illustrate the summed ripple numbers across time (bin size = 0.05 sec).

Page 10, line 259:

*…(i.e., 1100 – 1250 ms after reminder cue onset; also see Supplementary Fig. 17; see Supplementary Fig. 18 for exemplary raster plots of TMR ripples during high and low SO-spindle power trials)…*

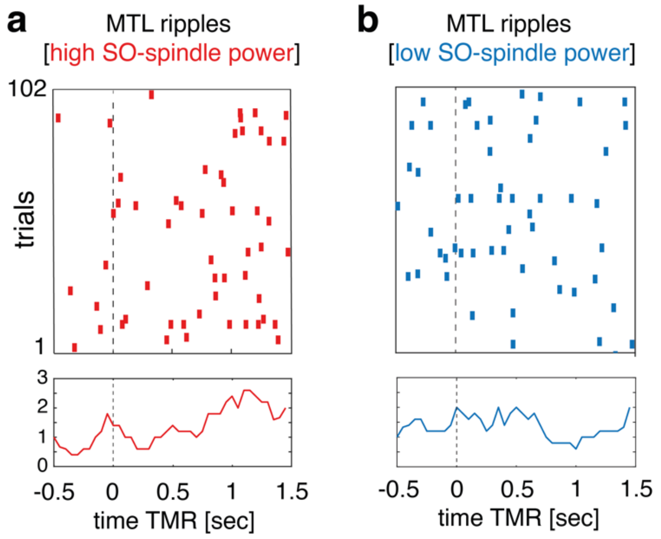


Supplementary Figure 18. (a) Exemplary raster plots for NREM sleep MTL ripples during high (red) and (b) low SO-spindle power trials. Bottom inserts illustrate the summed ripple numbers across time (bin size = 0.05 sec).

5) The authors should cite the papers mentioned above as they have direct relevance to these results, showing ripple-mediated memory reactivation during wakefulness.

Response: We cite now the suggested papers both in the results section and in the discussion.

Page 8, line 199:

*…Given the ascribed role of MTL ripples in memory retrieval [32–37], we assessed whether ripple rates would differ between remembered and not-remembered trials…*

Discussion, page 13, line 367:

*...In current models of memory consolidation, ripples are generally considered to be electrophysiological markers of memory reactivation, as they have been suggested to trigger retrieval-related memory reactivation [44] and the reprocessing of memories during sleep [1,20,45]. To date, however, even though the role of ripples in memory retrieval has been established in humans [32–37], direct evidence for a core contribution of ripples to sleep's memory function has been lacking in humans...*

6) The method employed for ripple detection may not be optimal. It is advisable for the authors to thoroughly review relevant intracranial studies on human hippocampal ripples published within the last five years (including the papers mentioned above), and subsequently fine-tune their methods accordingly. For instance, it is recommended to use a more stringent ripple detection threshold of 3-4 SD and exclude candidate ripples that occur near inter-ictal epileptic discharges and transient muscular artifacts that appear on the EMG electrode, rather than excluding 36% of the trials due to putative epileptiform activity. The authors seem to discard valuable information by implementing this stringent data selection criterion (i.e., effectively excluding ~36% of trials). Do the results hold true when trials are not subjected to this particular selection process?

Response: In the course of revising the manuscript we realized that the numbers provided in the methods section concerning artifact rejection were incorrect (page 20, line 650: 36.14 ± 5.51 % of all trials, $N_{allTrials}$ = 633 ± 26.48). Out of all TMR trials (N = 561.30 ± 22.49, 26.71 ± 5.24 % were discarded, while 29.03 ± 4.68 % of all retrieval-related trials (N = 256.8 ± 7.24) were rejected du to epileptic activity. We corrected the numbers in the methods section and apologize for this mistake.

That said, we now assessed the extend to what our results were dependent on the applied detection threshold (2 SD) and the artifact rejection regime in place. We repeated the ripple triggered classification analysis (Fig. 5d) using (i) a threshold of 3 SD for ripple detection and (ii) based the analysis on raw data without artifact rejection. Following this rationale 49.86 ± 6.06 ripples were detectable in 308 ± 17.05. Importantly, when training a classifier on the retrieval data [–0.5 to 1] and testing it on intracranial EEG segments centered around spindle-locked MTL ripples, a highly similar result, with reference to our original analysis, became apparent. We found a ripple - locked cluster of significant above-chance classification from –30 to 150 ms relative to ripple centers ($p$ = 0.032, corrected for multiple comparisons across time, associative retrieval time-window [-110 to 220 ms]. We do believe that this important control analysis underscores the reliability of our results. We added the figure to the Supplemental (Supplementary Figure 27) and refer to it in the main text (page 22, line 756).

*...(for cross-classification based on raw, non artifact-rejected data, where ripples were detected using a threshold of 3 SD, see Supplementary Fig. 27)...*

-- Ripple triggered classification [detection threshold 3SD; no artifact rejection] --

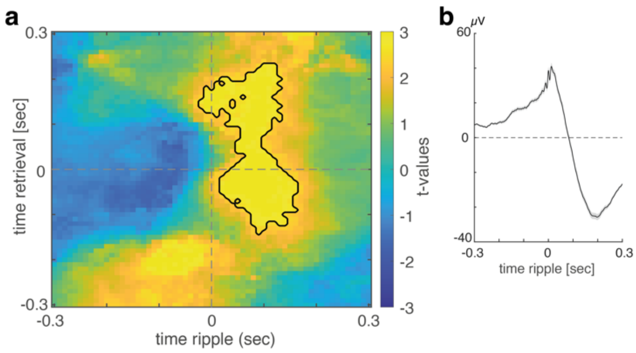


Supplementary Figure 27. (a) Classification procedure based on raw data (no artifact rejection), where ripples were detected using a threshold of 3 SD. Head orientation-related brain patterns (left vs. right) were decodable during the presence of spindle-locked MTL ripples (contour lines indicate the extent of the significant cluster, $p = 0.032$ corrected; color range (blue to yellow) represents t values). (b) Ripple-triggered grand average of all detected ripples (7 patients, 14 contacts; locked to maximal negative amplitude) during TMR (-.5 to 1.5 seconds; 49.86 ± 6.06 ripples in 308 ± 17.05 trials).

7) Critical point: the authors should extend the analysis in Figure 3e also to the awake retrieval phase. Does retrieval exhibit a comparable increase in ripple rate in response to the associative cue, as shown by Vaz et al. (2019)? If so, will the authors find stronger ripples activation for correctly remembered items? This analysis will substantially strengthen the link between the detected ripples and the mnemonic process.

Response: We thank the Reviewer for this intriguing comment. Following the Reviewers suggestion, we now detected ripples during retrieval trials (-0.5 to 1 seconds in relation to the onset of the associative memory prompt; please see below for an illustration of the ripple-triggered grand average of all detected ripples during retrieval and the corresponding time-frequency representation). We next computed the ripple rate (events / second) for remembered and not remembered trials, respectively. In line with previous findings (e.g., Vaz et al., 2019) successful retrieval was associated with an increase in the number of MTL ripples ($p = 0.047$, time-window of significance: 0.4 to 0.5 seconds after memory prompt onset; corrected across time). Please note that ripple rates during remembered and not-remembered trials descriptively differed already during the baseline window (-.5 to 0 sec), with higher ripple rates for remembered trials. This difference, even though non-significant, probably is related to the specifics of the administered retrieval test, where associative memory was always tested after the old / new judgements during the preceding recognition test.
Taken together, we do think that these additional results significantly broaden the scope of our work. We added the ripple related retrieval results to Figure 3 and refer to them in text.

Results (page 8, line 199):

*…Given the ascribed role of MTL ripples in memory retrieval [32–36], we assessed whether ripple rates would differ between remembered and not-remembered trials. First, ripples were extracted (7 patients, 14 contacts) based on established criteria [18] (see Methods for details; see Fig. 3c and 3d for ripples related EPRs and time-frequency representations). Then peri-event histograms (bin size = 50 ms) of ripple events time-locked to the onset of the associative retrieval prompt were calculated, specifically for remembered and not-remembered trials. Ripple rates (events per second) differed significantly between conditions (p = 0.047; corrected for multiple comparisons across time), with higher MTL ripple rates for remembered as compared to not-remembered trials (i.e., 400 – 500 ms after the onset of the associative retrieval prompt). It should be noted that ripple rates during remembered and not-remembered trials descriptively differed already during the baseline window (-.5 to 0 sec), with higher ripple rates for remembered trials. This non-significant difference is probably related to the fact that*

*associative memory was always test right after old / new judgements, hence retrieval processes were not exclusively triggered by the associative memory prompt onset…*

Discussion (page 13, line 367):

*…In current models of memory consolidation, ripples are generally considered to be electrophysiological markers of memory reactivation, as they have been suggested to trigger retrieval-related memory reactivation [44] and the reprocessing of memories during sleep [1,20,45]. To date, however, even though the role of ripples in memory retrieval has been established in humans [32–37], direct evidence for a core contribution of ripples to sleep's memory function has been lacking in humans…*

Page 13, line 384:

*…One intriguing question is whether all ripples are related to memory reactivation. Our data suggest that, while ripple rate distinctly peaked during successful remembering in an early time-window, only a fraction of NREM ripples, specifically those coupled to cortical spindles, were associated with ~~driving~~ the decodability of prior retrieved head-orientations (Fig. 4b and Supplementary Fig. 21). Spindles are well known to group ripples in the MTL [19,21–23] (Fig. 4a)…*

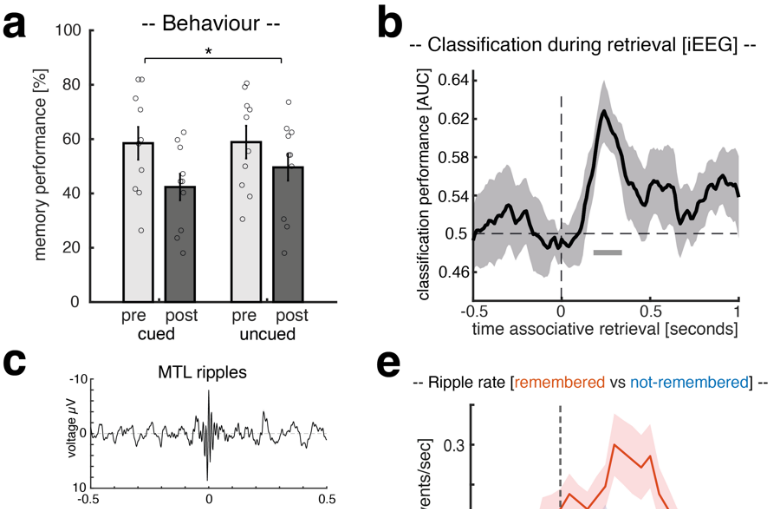


Fig.3 intracranial EEG study: retrieval. (a) Behavioral results for both experimental sessions pre- (light gray) and post-sleep (dark gray), separated into cued and uncued trials. Bar graphs show mean (±SEM) percentage of recalled head orientations. Dots indicate individual memory performance of participants (N = 10). The star denotes the significant interaction (pre vs. post x cued vs. uncued) as derived from a repeated measures ANOVA ($F_{1,9}$ = 8.28; *p* = 0.018). (b) Later cued head orientation (left vs. right) could be reliably decoded (above chance) from the retrieval data, starting around 190 ms after the onset of the associate prompt (the black solid line indicates decoding performance (±SEM)). The horizontal dashed line indicates chance level performance (i.e., 0.5). The vertical solid line indicates the onset of associative retrieval trials (time = 0). The lower horizontal gray line shows the temporal extent of significant decoding results as derived from a dependent-samples t-test (two-sided, *p* = 0.019, cluster corrected across time). (c) Ripple-triggered grand average of all detected ripples (7 patients, 14 contacts; locked to maximal negative amplitude) during retrieval (-.5 to 1.5 seconds ). (d) Time-frequency-representations of ripple-locked MTL data segments. (e) Ripple rates (events per second) for remembered (red) and not-remembered (blue) trials. Ripple rates differed significantly between conditions (*p* = 0.047; corrected), with MTL ripples peaking during remembered trials (0.4 to 0.5 seconds in relation to the memory prompt onset).

8) Critical point: the authors should add a figure showing the cross-classification performance using the broadband high-gamma intracranial signal. This signal reflects neuronal activation more accurately and directly, compared to the raw iEEG signal. It is crucial to investigate whether the timing of reactivation remains consistent between the raw iEEG and high-gamma signals, as this has significant implications for interpreting the results.

Response: Following the Reviewers suggestion we repeated both the classification during retrieval (Fig. 3b) as well as the cross-classification (training the classifier on retrieval data and testing on TMR data; Fig 4b) on basis of broadband high -gamma iEEG data (50 – 140 Hz). Consistent with our previous result, head orientation-related signatures were decodable during retrieval with above-chance classification accuracy emerging around the onset of the associative memory prompt (time window: -80 ms to 800 ms; peak at 360 ms; *p* = 0.004, corrected for multiple comparisons across time). Also for the high-gamma based cross-classification analysis results revealed significant above-chance classification from 910 to 1480 ms relative to TMR onset (*p* = 0.022, corrected for multiple comparisons across time), emerging during the presence of sleep spindles (associative retrieval time-window: 80 to 800ms). While the cluster of significance highly overlapped with the one described in our main analysis (960 to 1410 ms relative to TMR onset, associative retrieval time-window: -150 to 200 ms) it has to be noted that a heightened amount of retrieval-related activity was decodable during TMR in the gamma-based analysis. To preserve the comparability with the scalp EEG related results (were high-gamma related analyses are not feasible) we decided to remain with our original result in the main text and corresponding Fig. 4. However, we added the new results to the Supplemental (Supplementary Figure 9) and refer to it in the main text (page 8, line 193).

*…(peak at 250 ms; p = 0.019, corrected for multiple comparisons across time, see Fig. 3b; for classification results based on broadband high-gamma data see Supplementary Fig. 9…*

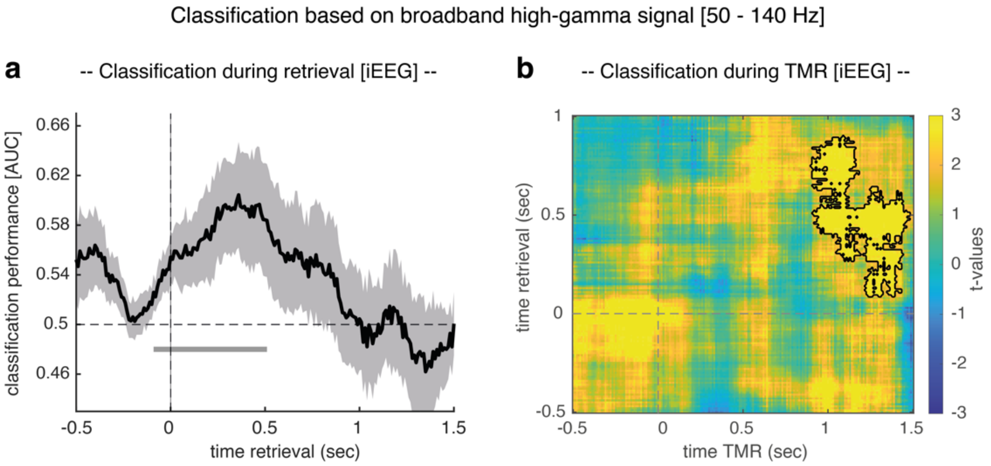


Supplementary Figure 9. (a) Later cued head orientation (left vs. right) could be reliably decoded (above chance) from the retrieval data filtered in the high-gamma range (50 – 150 Hz), starting around 80 ms after the onset of the associate prompt (the black solid line indicates decoding performance (±SEM across participants)). The horizontal dashed line indicates chance level performance (i.e., 0.5). The vertical solid line indicates the onset of associative retrieval trials (time = 0). The lower horizontal gray line shows the temporal extent of significant decoding results as derived from a dependent-samples t-test (two-sided, *p* = 0.019, cluster corrected across time). (b) Head orientation-related brain patterns (left vs. right) were decodable during TMR based on high-gamma filtered data (50-150 Hz; contour lines indicate the extent of the cluster, *p* = 0.022 corrected; color range (blue to yellow) represents t values against chance level performance.

9) The authors should run the high-gamma analysis of ripple-triggered activity both during TMR trials and awake retrieval trials. If ripples indeed play a role in re-activating memory associations, they should do so both during sleep and awake retrieval (as shown by previous

studies). The authors should examine if the similarity between the cortical peri-ripple patterns induced by TMR and those induced by successful memory retrieval, is significantly higher compared to peri-ripple patterns induced by foil presentation (where memory associations do not exist).

Response: We repeated the retrieval-related decoding procedure with high-gamma filtered (50 to 150 Hz) iEEG segments centered around MTL ripples, specifically for remembered and not-remembered trials (we deviated from the Reviewers suggestion to contrast remembered with foil-related peri-ripple patterns, since whenever participants made “new” responses during recognition testing, they immediately moved on to the next trial, hence omitting the possibility to acquire foil related activity after the onset of the associate memory prompt). That said, 2 patients had to be excluded from this analysis due to a too small number of detected ripples (N < 10) in one of the relevant conditions (left-right, remembered – not-remembered), leading to 5 patients, exhibiting contacts in the MTL entering the analysis. As shown below, classification performance was generally enhanced for remembered as compared to not-remembered stimuli (peak of difference: -90 ms in relation to ripple center). However, this difference did not reach significance ($p > 0.05$), potentially due to low power. In addition, we repeated the cross-classification procedure locked to ripples during TMR on basis of gamma-filtered data (50 – 150Hz). A classifier was trained on the pooled associative retrieval data, but the resulting training weights were applied on intracranial EEG segments centered around MTL ripples. Consistent with the results of our main analysis on raw iEEG data we found a ripple - locked cluster of significant above-chance classification from – 300 to 140 ms relative to ripple centers ($p = 0.015$, corrected for multiple comparisons across time, associative retrieval time-window [-160 to 170 ms]). We added these new results to the Supplemental (Supplementary Figure 12) and refer to them in the main text (page 8, line 209).

*…(p = 0.047; corrected for multiple comparisons across time), with higher MTL ripple rates for remembered as compared to not-remembered trials (i.e., 400 – 500 ms after the onset of the associative retrieval prompt; for classification results based on ripple-triggered broadband high-gamma data see Supplementary Fig. 12)…*

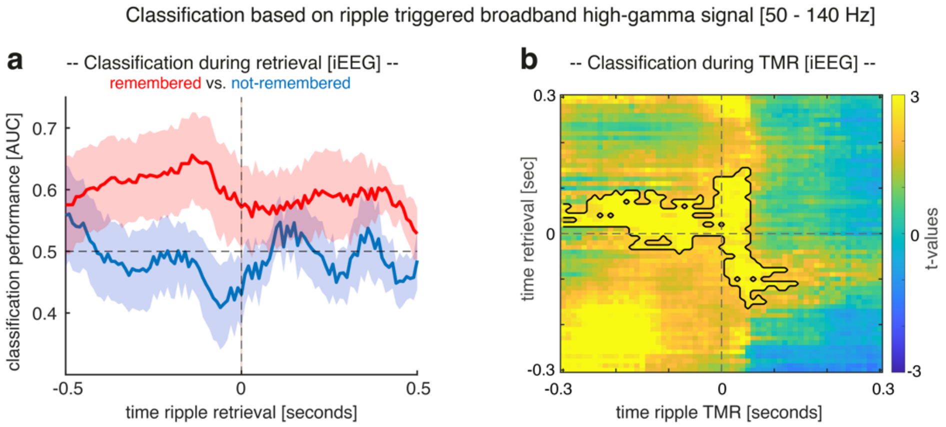


Supplementary Figure 12. (a) Retrieval-related classification of head orientations on basis of high-gamma filtered (50 to 150 Hz) iEEG segments centered around MTL ripples, specifically for remembered and not-remembered trials. While classification performance was generally higher for remembered as compared to not-remembered stimuli (peak of difference: -90 ms in relation to ripple center), this difference did not reach significance (p > 0.05). (b) Head orientation-related brain patterns (left vs. right) were decodable during the presence of MTL ripples based on high-gamma filtered iEEG data (50 – 150 Hz, contour lines indicate the extent of the significant cluster, $p = 0.015$ corrected; color range (blue to yellow) represents t values).

**General comments:**

10) Overall, the findings don't conclusively establish whether the observed reactivation genuinely indicates a mnemonic process or whether it primarily pertains to activity linked with cue identity. Put differently, can the authors discern whether the reactivated representations are connected to memory representation, head orientation, or the identity of auditory cues? Specifically, it would be beneficial if the authors could show that a classifier trained on encoding trials (when subjects actually moved their head), is able to decode the same head-orientations during retrieval. (If this analysis proves inconclusive, it is advisable for the authors to temper their claims in the discussion regarding reactivation of representational content that is specifically tied to "head-orientation"; currently the results do not conclusively show that)

Response: We want to thank the Reviewer for this important comment. First, it is important to note that it is not conceivable that our decoding results were impacted by neural activity associated with the processing of auditory cues. The sound cues were not presented during retrieval, hence safely excluding that they influenced either the within-retrieval classification or the cross-classification (where the retrieval data served as training data). That said, the Reviewer is correct that the data at hand does not necessarily imply that actual head orientation-related information was reactivated. Hence, we followed the Reviewer's suggestion and trained a classifier on the encoding data (of the iEEG study, where movement related artifacts are less problematic) and tested it on the retrieval data.

Specifically, the encoding data were first segmented with respect to the image presentations on the 4 monitors (-0.5 to 1 sec). Next, encoding data and data belonging to remembered retrieval trials were z-scored across trials and collapsed. PCA was applied to the pooled encoding-retrieval data and the first 30 principal components were retained. Encoding and retrieval data were smoothed using a running average window of 150 ms. A classifier was then trained for every time point in the encoding data and applied on every time point during retrieval. As for the within retrieval decoding, the classifier was trained and tested to discriminate between the later cued head orientations (i.e., one left sided and one right sided head orientation; see Targeted Memory Reactivation for details). No cross-validation was required since encoding and retrieval datasets were independent.

As shown below, results revealed significant above-chance classification from -250 to 200 ms relative to memory retrieval prompt onset ($p < 0.001$, corrected for multiple comparisons across time; encoding-related time-window: -250 to 500 ms). The fact that decodability preceded the onset of image presentations during encoding (and hence actual head-movements) most likely reflects that auditory cues predicted the upcoming relevant head orientations and participants already prepared the execution of the movement. Please note, that, again, processing of the auditory cues (which were presented 600ms before image onset) did not influence these results as they were not part of the retrieval. In sum, we do believe that these results strongly support our interpretation that head orientation-related signatures were reactivated during retrieval. We added this information to the main text (page 8; line 194) and included the Figure in the Supplemental (Supplemental Fig. 10).

*…(peak at 250 ms; $p = 0.019$, corrected for multiple comparisons across time, see Fig. 3b; for classification results based in broadband high-gamma data see Supplementary Fig. 9; for results concerning the classification of encoding-related brain patterns during retrieval see Supplementary Fig. 10)…*

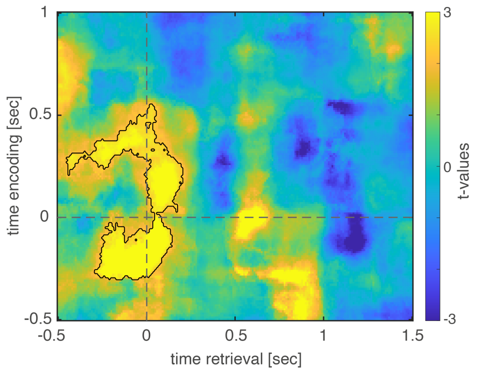


Supplementary Figure 10. Encoding-related brain patterns (locked to image presentation; later cued left vs. right head orientations) were decodable during remembered retrieval trial (contour lines indicate the extent of the cluster, $p < 0.001$ corrected across time; color range (blue to yellow) represents t values against chance level performance), indicating that indeed head orientation-related brain patterns were picked up by the classifier.

11) Line 416: “we found a positive correlation between memory confidence in the pre-sleep memory test and the effect of TMR”: this is an important finding. As a follow-up analysis, it would be beneficial for the authors to investigate whether the behavioral outcomes and their correlation with the TMR effect become more pronounced when excluding "guesses." These would be trials where subjects reported very low confidence levels (<3). If subjects do not remember specific items during the pre-sleep test, it is not expected that TMR would substantially enhance their memory. The potential for memory enhancement through TMR would primarily come into play when a memory trace already exists.

Response: For the correlation between the confidence rating and in the pre-sleep memory test and the effect of TMR shown in Supplementary Fig. 23 (please see below) only high confidence trials were included (confidence rating = 4). We think that this ultimately corresponds to what the Reviewer suggests, by including only trials where it seems clear that a memory trace exists. The results that those high confidence trials profited from TMR and potentially were driving the decoding results are very much in line with this interpretation.

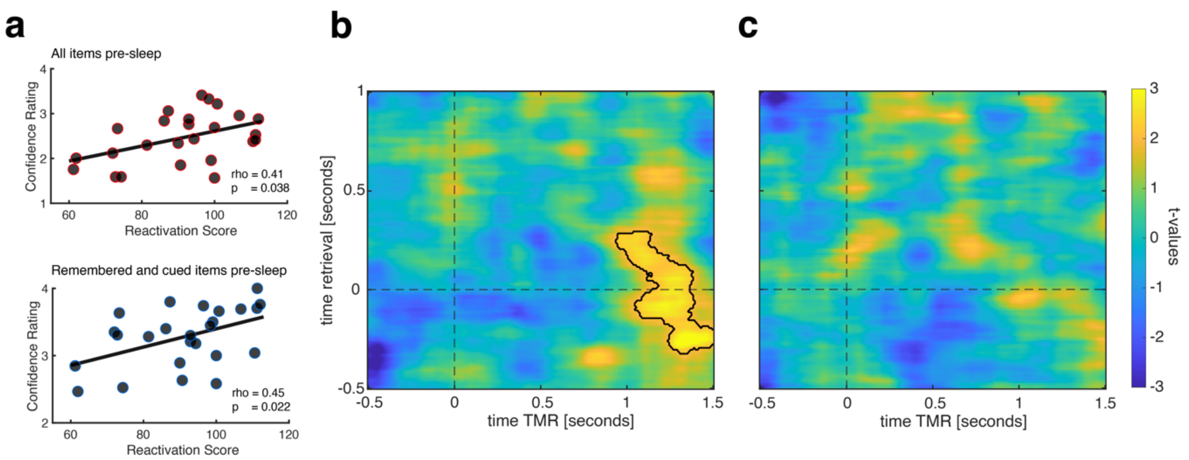


**d**

| | Pre | Post | Pre-rem | Post-rem | Pre high conf. rem | Post high conf. rem |
|---|---|---|---|---|---|---|
| Confidence | 2.5 ± 0.5 | 2.3 ± 0.6 | 3.2 ± 0.3 | 3.3 ± 0.2 | 54.39 ± 3.97 % | 55.53 ± 3.99 % |

Supplementary Figure 23. Relationship of confidence ratings and memory reactivation in the scalp EEG study. In each retrieval trial, participants indicated how confident they were with their head orientation decision (scale from 0 corresponding to very uncertain to 4, very certain). (a) Participants' average confidence ratings across all trials correlated positively with the behavioral impact of TMR (i.e., reactivation score: relative difference from pre- to post-sleep for cued items - relative difference from pre- to post- sleep for uncued items * 100)/ pre-sleep remembered items; rho = 0.41; $p$ = 0.038). Likewise, participants' average confidence ratings across all remembered and cued trial correlated positively with the behavioral impact of TMR (i.e., reactivation score: relative difference from pre- to post-sleep for cued items - relative difference from pre- to post- sleep for uncued items * 100)/ pre-sleep remembered items; rho = 0.41; $p$ = 0.038). These results suggest that highly confident participants rather benefitted from TMR, while less confident participants exhibited a detrimental effect of TMR. (b) Classification using only high confidence trials (confidence rating = 4) as training data. Retrieval-related brain patterns (left vs. right head orientations) were reliably decodable when the decoder was trained on high confidence trials (contour lines indicate the extent of the significant cluster, $p$ = 0.025 corrected; color range (blue to yellow) represents t values against chance level performance (i.e., 0.5)). (c) Classification using lower confidence trials (confidence rating < 4) as training data. Retrieval-related brain patterns (left vs. right head orientations) were not decodable when the decoder was trained on lower confidence trials ($p$ = 0.96 corrected; color range (blue to yellow) represents t values against chance level performance (i.e., 0.5)). (d) Descriptives of confidence ratings: Means and SEM of confidence ratings of all pre-sleep trials [Pre], all post-sleep trials [Post], all remembered pre-sleep trials [Pre-rem] and all remembered post-sleep trials [Post-rem]. Percentage of high confidence trials of all remembered trials in the pre-sleep memory test [Pre high conf. rem] and percentage of high confidence trials of all remembered trials in the post-sleep memory test [Post high conf. rem].

12) Multivariate analysis: how did you choose to retain 30 principal components? how much variance these components explain? Please include the ERPs associated with the two head-orientation classes for readers who want to appreciate the quality of the EEG signal.

<u>Response</u>: The rationale of retaining 30 components was based on the existing literature (e.g.; Brouwer & Heeger, 2009). In the scalp EEG study, the 30 components explained 96.83 ± 0.2 % of the variance, while 97.60 ± 0.17 % in the iEEG study. We added this information to the Methods section (page 21, line 687).

*… PCA was applied to reduce dimensionality and limit over-fitting (PCA) and the first 30 principal components were retained for further analysis (explaining 96.83 ± 0.2 % of the variance in the scalp EEG study and 97.60 ± 0.17 % in the iEEG study)…*

In addition, we added the ERPs associated with the head orientation classes (for both the scalp and the iEEG group) to the Supplemental (Supplemental Fig. 25; also see below) and refer to it in text (page 20, line 644).

*...The retrieval data were additionally subjected to an independent component analysis (ICA) and ICA components associated with eye blinks and eye movements were identified and rejected (for ERPs related to left and right stimulus categories for retrieval and TMR see Supplementary Fig. 25)...*

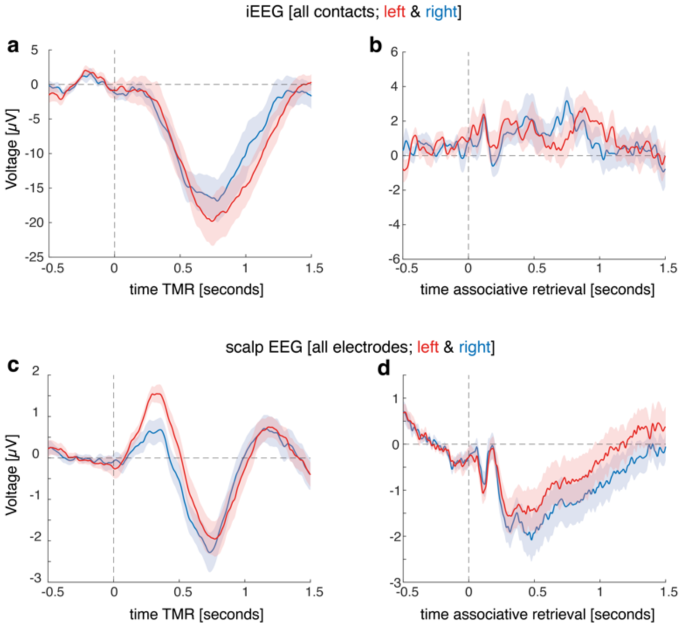


Supplementary Figure 25. (a + b) Event-related potentials (ERPs, mean ± SEM across participants) for the iEEG study with regards to TMR (a) and the associative memory retrieval (b). The red waveform corresponds to ERPs associated with cues that were associated with left-sided head-orientations during encoding. The blue waveform corresponds to ERPs associated with cues that were associated with right-sided head-orientations during encoding. (c + d) ERPs (mean ± SEM across participants) for the scalp EEG study with regards to TMR (c) and the associative memory retrieval (d). The red waveform corresponds to ERPs associated with cues that were associated with left-sided head-orientations during encoding. The blue waveform corresponds to ERPs associated with cues that were associated with right-sided head-orientations during encoding.

13) Where is the iEEG reactivation effect localized? The authors are encouraged to provide a cortical map illustrating the spatial distribution of electrodes that play a significant role in the classification result.

Response: To answer the question where iEEG reactivation effects are localized, we followed a leave-one-region-out approach with regards to cross-classification. Specifically, we repeated the cross-classification 5 times and in each iteration iEEG contacts belonging to one region of interest (i.e., frontal, parietal, temporal, occipital, MTL) were excluded from the analysis. Results revealed that excluding temporal, occipital and MTL contacts did not affect the outcomes of the cross-classification procedure, given that head orientation-related activity was decodable during TMR (temporal: $p = 0.029$, occipital: $p = 0.026$, MTL: $p = 0.021$). Interestingly, when excluding frontal or parietal contacts no cluster of significant above chance classification became apparent (frontal: $p = 0.054$, parietal: $p = 0.052$), indicating that frontal and parietal areas might have played a more prominent role with regards to sleep based memory reactivation, even though the interpretation of p-values close to $p = .05$ warrants caution. However, the present pattern of results not only matches results of our scalp EEG based searchlight procedure, which indicated that fronto-parietal networks might have been implicated in TMR-induced memory reactivation, but also findings in rodent models were replay activity has been described in prefrontal and parietal cortex (e.g., Peyrache et al., 2009; Wilber et al., 2017).
However, it has to be noticed that the results of the present analysis have to be interpreted with caution, as the number of contacts per region differed across patients. To visualize the obtained results, we summed the t-values within the cluster exhibiting the smallest p-value (please see below). Hence, smaller numbers (color-coded in green) represent areas that

putatively contributed to the detection of memory reactivation, since excluding them led to non-significant results. We added the result to the Supplemental (Supplemental Fig. 20) and refer to it in the main text (page 10; line 279).

*…These results indicate that (i) TMR-induced reactivation is related to remembered head orientations and that (ii) reactivation was putatively mediated by SO-spindle and ripple activity (for a localization of the cross-classification result see Supplementary Fig. 20)…*

Localizing Memory reactivation in iEEG

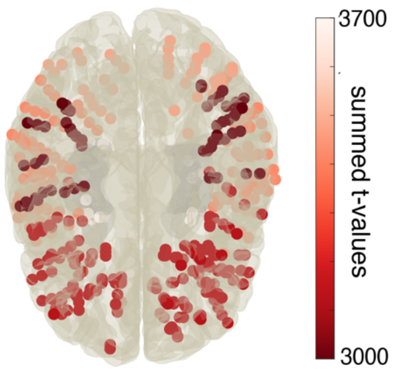


Supplementary Figure 20. To estimate the contribution of different brain areas to the cross-classification results (Fig. 4e), a leave-one-region-out approach was conducted. Specifically, we repeated the cross-classification 5 times and in each iteration iEEG contacts belonging to one region of interest (i.e., frontal, parietal, temporal, occipital, MTL) were excluded from the analysis. Results revealed that excluding temporal, occipital and MTL contacts did not affect the outcomes of the cross-classification procedure, given that head orientation-related activity was still decodable during TMR (temporal: $p = 0.029$, occipital: $p = 0.026$, MTL: $p = 0.021$). When excluding frontal or parietal contacts, no cluster of significant above chance classification became apparent (frontal: $p = 0.054$, parietal: $p = 0.052$), indicating that these areas might have played a necessary role with regards to sleep based memory reactivation. It has to be noticed, that the results of the present analysis have to be interpreted with caution, as the number of contacts per region differed across patients. To visualize the obtained results, we t-values within the cluster exhibiting the smallest p-value were summed. Hence, smaller numbers (color-coded in green) represent areas that putatively contributed to the detection of memory reactivation, since excluding them led to non-significant results.

## Minor points:

14) Line 39: "establishing a role of sharp wave-ripples for memory reactivation" - the role of ripples in memory reactivation has already been fairly established in previous works, especially during awake retrieval; the present work only extends this to sleep and specifically to ripples that are nested within spindles; this work is certainly not the first to show that ripples are related to memory reactivation (e.g., Zhang et al. 2018).

Response: We apologize for not being precise here. We now specify that our results suggest a role of ripples for memory reactivation during human NREM sleep (page 3; line 40).

*…Leveraging direct access to medial temporal lobe (MTL) electrophysiology in epilepsy patient, we show that spindle-locked ripples are instrumental for memory reactivation to unfold during human sleep, establishing a role of sharp wave-ripples for memory reactivation in human NREM sleep…*

15) Line 75: "Head orientation-related activity is reactivated" unless the authors show that the reactivation was specifically related to head orientation representations, the authors should revise their claims and refer to this activity as memory-prompt related activity.

Response: We think that the results presented above (response to Comment 10) indicating that encoding-related activity was decodable during retrieval permit the used language.

16) Line 84: the authors refer to a figure that does not exist (Fig 1d).

Response: Thanks for spotting this. We corrected the mistake.

*… We performed multivariate classification (linear discriminant analysis; LDA) on these data (Fig. 1c)…*

17) Line 115-116: what is meant by "please note that statistical tests were done for illustrative purposes only" are all the statistics in this figure are for "illustrative purposes"?

Response: We apologize for the ambiguity. This sentence only refers to the statistical test with regards to the searchlight procedure, as the statistics were informed by the outcomes of the standard classification approach, which might qualify as double dipping (i.e., time windows that entered statistics were based on the previous outcomes). We rephrased the sentence to make clear that just for the searchlight procedure statistics was done for illustrative purposes.

*…(please note that statistical tests with regards to the searchlight procedure were done for illustrative purposes only)…*

18) Line 468: typo, remove the word "and".

Response: We corrected the mistake.

19) Line 342, 385: the authors should also cite Rothchild et al 2016 ((Oyarzún et al., 2017; Antony et al., 2018; Witkowski et al., 2021; Joensen et al., 2022)) showing TMR in rodents. The authors should discuss their results considering this paper, which suggests that auditory TMR facilitates generation of memory-related ripples by biasing hippocampal activity.

Response: We now added a section, discussing our results in light of the findings by Rothschild and colleagues (2017, 2019).

Page 13, line 375:

*…Interestingly, TMR studies in rodents have not only shown that auditory cues induce memory reactivation in the hippocampus [46,47], but that cortical activity elicited by memory-related cues, predicts hippocampal ripple activity, which in turn biases post-ripple activity in cortical areas, suggesting a loop-like information flow around ripple occurrence [46,48]. Our result that ripples in the human MTL were tightly associated with TMR elicited memory reactivation is in line with the notion that auditory TMR might facilitate generation of memory-related ripples by biasing hippocampal activity. However, our current data remain agnostic as to whether a cortical-hippocampal-cortical loop of information exchange underlies sleep-related memory consolidation…*

20) Lines 652, 690: "Fig 3c" should be fig 3d

Response: We corrected the mistake.

21) Fig 1d: correct “TFR” to TMR.

Response: We assume the Reviewer is referring to Fig. 2d (correlation between classification performance and TMR triggered power). TFR refers to the fact that power was extracted from time-frequency representations. We made that point now clear in text (page 6, line 145).

*…To address this question, we correlated, across participants, TMR triggered power as obtained by time-frequency representations (TFR; averaged across the cluster shown in Fig. 2a) and levels of mean classification performance (averaged across the cluster shown in Fig. 2b)…*

22) In all figures: please indicate what the error bars represent (SEM across subjects/patients/electrodes/trials?)

Response: We indicate now in each figure what the error bars refer to.

References:


Andrillon T, Poulsen AT, Hansen LK, Léger D, Kouider S (2016) Neural Markers of Responsiveness to the Environment in Human Sleep. J Neurosci 36:6583–6596.

Andrillon T, Pressnitzer D, Léger D, Kouider S (2017) Formation and suppression of acoustic memories during human sleep. Nat Commun 8:179.

Antony JW, Cheng LY, Brooks PP, Paller KA, Norman KA (2018) Competitive learning modulates memory consolidation during sleep. Neurobiology of Learning and Memory 155:216–230.

Cairney SA, Guttesen AÁV, El Marj N, Staresina BP (2018) Memory Consolidation Is Linked to Spindle-Mediated Information Processing during Sleep. Curr Biol 28:948-954.e4.

Cowie RJ, Robinson DL (1994) Subcortical contributions to head movements in macaques. I. Contrasting effects of electrical stimulation of a medial pontomedullary region and the superior colliculus. Journal of Neurophysiology 72:2648–2664.

Elsley JK, Nagy B, Cushing SL, Corneil BD (2007) Widespread presaccadic recruitment of neck muscles by stimulation of the primate frontal eye fields. J Neurophysiol 98:1333–1354.

Joensen BH, Harrington MO, Berens SC, Cairney SA, Gaskell MG, Horner AJ (2022) Targeted memory reactivation during sleep can induce forgetting of overlapping memories. Learn Mem 29:401–411.

Kouider S, Andrillon T, Barbosa LS, Goupil L, Bekinschtein TA (2014) Inducing task-relevant responses to speech in the sleeping brain. Curr Biol 24:2208–2214.

Lewis PA, Bendor D (2019) How Targeted Memory Reactivation Promotes the Selective Strengthening of Memories in Sleep. Curr Biol 29:R906–R912.

Ngo H-VV, Staresina BP (2022) Shaping overnight consolidation via slow-oscillation closed-loop targeted memory reactivation. Proc Natl Acad Sci U S A 119:e2123428119.

Oyarzún JP, Morís J, Luque D, Diego-Balaguer R de, Fuentemilla L (2017) Targeted Memory Reactivation during Sleep Adaptively Promotes the Strengthening or Weakening of Overlapping Memories. J Neurosci 37:7748–7758.

Peyrache A, Khamassi M, Benchenane K, Wiener SI, Battaglia FP (2009) Replay of rule-learning related neural patterns in the prefrontal cortex during sleep. Nat Neurosci 12:919–926.

Schreiner T, Petzka M, Staudigl T, Staresina BP (2021) Endogenous memory reactivation during sleep in humans is clocked by slow oscillation-spindle complexes. Nat Commun 12:3112.

Schreiner T, Staudigl T (2020) Electrophysiological signatures of memory reactivation in humans. Philos Trans R Soc Lond B Biol Sci 375:20190293.

Schurger A, Hu P “Ben,” Pak J, Roskies AL (2021) What Is the Readiness Potential? Trends in Cognitive Sciences 25:558–570.

Shibasaki H, Hallett M (2006) What is the Bereitschaftspotential? Clin Neurophysiol 117:2341–2356.

Sparks DL, Freedman EG, Chen LL, Gandhi NJ (2001) Cortical and subcortical contributions to coordinated eye and head movements. Vision Res 41:3295–3305.

Wang B, Antony JW, Lurie S, Brooks PP, Paller KA, Norman KA (2019) Targeted Memory Reactivation during Sleep Elicits Neural Signals Related to Learning Content. J Neurosci 39:6728–6736.

Wilber AA, Skelin I, Wu W, McNaughton BL (2017) Laminar Organization of Encoding and Memory Reactivation in the Parietal Cortex. Neuron 95:1406-1419.e5.

Witkowski S, Noh SM, Lee V, Grimaldi D, Preston AR, Paller KA (2021) Does memory reactivation during sleep support generalization at the cost of memory specifics? Neurobiol Learn Mem 182:107442.

REVIEWERS' COMMENTS

Reviewer #1 (Remarks to the Author):

The authors have fully addressed my concerns, and have also added some nice new analyses and discussion paragraphs in response to the other reviewers.

* Minor comment: Supplementary Figure 20 - I believe the authors meant "color-coded in red" (rather green)

Sincerely

Reviewer #2 (Remarks to the Author):

First, I'd like to congratulate the authors on an impressively thorough revision. I think it's basically a complete paper, though I'm left feeling like the findings are disconnected. To that point, I've one comment I hope they consider followed by a few minor points.

Major comment:

I'm still left feeling like these are almost two papers, or at least that there's a disconnect between the neural and behavioral results in the main paper. Behaviorally, they see TMR impairments. Neurally, they see a nice alignment of classification with SOs, spindles, and ripples, but no effects on behavior. I think the authors should consider moving what is now in Supp Fig 23 into the main paper, because that at least offers a plausible story linking the behavioral and neural results (that is, the possibility that high confidence items benefit (and show neural reactivation!) while low confidence items worsen (and have no reactivation). It does not bother me personally that they would not be able to replicate this analysis in the iEEG study because confidence was not measured.

Minor comments:

I believe the authors still have some causal language where correlational is warranted. Here are some suggestions:

Abstract: “SO-spindle activity promoted” -> “SO-spindle activity coincided with”

Abstract: “be necessary for” -> “correlate with”

Line 304: “facilitate” -> “coincide with”

I also think “ignites” in line 117 would be better as “elicits” or “induces”. Additionally, I do not see how the SO-spindle burst foreshadows relevant neural processing on lines 120-124 -- one could easily envision SO-spindle bursts occurring without relevant learning-related reactivation. I believe they can just cut the “foreshadowing” clause on lines 123-124.

Reviewer #3 (Remarks to the Author):

The authors have satisfactorily addressed my comments, congratulations for a nice and well-designed study

**Reviewer #1** (Remarks to the Author):

The authors have fully addressed my concerns, and have also added some nice new analyses and discussion paragraphs in response to the other reviewers.

*Response: Thank you for the positive feedback .*

* Minor comment: Supplementary Figure 20 - I believe the authors meant "color-coded in red" (rather green)

Sincerely

*Response: Changed accordingly.*

**Reviewer #2** (Remarks to the Author):

First, I'd like to congratulate the authors on an impressively thorough revision. I think it's basically a complete paper, though I'm left feeling like the findings are disconnected. To that point, I've one comment I hope they consider followed by a few minor points.

Major comment:
I'm still left feeling like these are almost two papers, or at least that there's a disconnect between the neural and behavioral results in the main paper. Behaviorally, they see TMR impairments. Neurally, they see a nice alignment of classification with SOs, spindles, and ripples, but no effects on behavior. I think the authors should consider moving what is now in Supp Fig 23 into the main paper, because that at least offers a plausible story linking the behavioral and neural results (that is, the possibility that high confidence items benefit (and show neural reactivation!) while low confidence items worsen (and have no reactivation). It does not bother me personally that they would not be able to replicate this analysis in the iEEG study because confidence was not measured.

*Response: We decided to add a dedicated paragraph to the results section, depicting the potential interactions between confidence ratings and memory reactivation.*

*Page 6, line 121:*

*Relationship between behavioral performance and memory reactivation*

*In an exploratory analysis (see Supplementary Fig. 7), we investigated the relationship of confidence ratings and memory reactivation in the scalp EEG study. We found a positive correlation between memory confidence in the pre-sleep memory test and the reactivation score (rho = 0.41; p = 0.038), which was also found when restricting the analysis to remembered and cued trials (rho = 0.43 p = 0.028; see Supplementary Fig. 7a). Restricting the classification of head orientations to only high confidence trials showed significant above-chance decoding of retrieval-related brain patterns during NREM sleep (p = 0.025, corrected for multiple comparisons across time see Supplementary Fig. 7b), a pattern highly similar to the pattern found when all trials were included (see Fig. 3b). In contrast, when restricting the classification of head orientations to low-confidence trials, retrieval-related brain patterns (left vs. right head orientations) were not decodable (see Supplementary Fig. 7c).*

Minor comments:
I believe the authors still have some causal language where correlational is warranted. Here are

some suggestions:
Abstract: “SO-spindle activity promoted” -> “SO-spindle activity coincided with”
Abstract: “be necessary for” -> “correlate with”
Line 304: “facilitate” -> “coincide with”

*Response: We changed the wording as suggested by the reviewer.*

I also think “ignites” in line 117 would be better as “elicits” or “induces”. Additionally, I do not see how the SO-spindle burst foreshadows relevant neural processing on lines 120-124 -- one could easily envision SO-spindle bursts occurring without relevant learning-related reactivation. I believe they can just cut the “foreshadowing” clause on lines 123-124.

*Response: The reviewer is correct in stating that SO-spindle bursts per se do not necessarily mirror relevant neural processing. However, we report that learning related cues induce enhanced activity in the SO-spindle range as compared to control cues (i.e., which were not part of the learning material). We do believe that the elevated levels of power in the SO-spindle range for learning related cues, indeed imply that some relevant processing was related to the presence of SO-spindles. This interpretation is not only in line with the current literature (e.g., Brodt et al, 2023), but is importantly further supported by our finding that power in the SO-spindle range was significantly correlated with decoding performance (Fig. 2d). In conclusion, we would oppose to omitting the sentence.*

**Reviewer #3** (Remarks to the Author):

The authors have satisfactorily addressed my comments, congratulations for a nice and well-designed study

*Response: Thank you for the positive feedback .*
